# Supplementary material for: Interferon gene therapy reprograms the leukemia microenvironment inducing protective immunity to multiple tumor antigens
Source: Nat Commun. 2018 Jul 24;9:2896. doi: 10.1038/s41467-018-05315-0 (PMC6057972; doi:10.1038/s41467-018-05315-0)
Supplement: Supplementary file 1 — Supplementary Information [file 41467_2018_5315_MOESM1_ESM.pdf]

Interferon gene therapy reprograms the leukemia microenvironment inducing protective immunity to multiple tumor antigens

Escobar et al.,

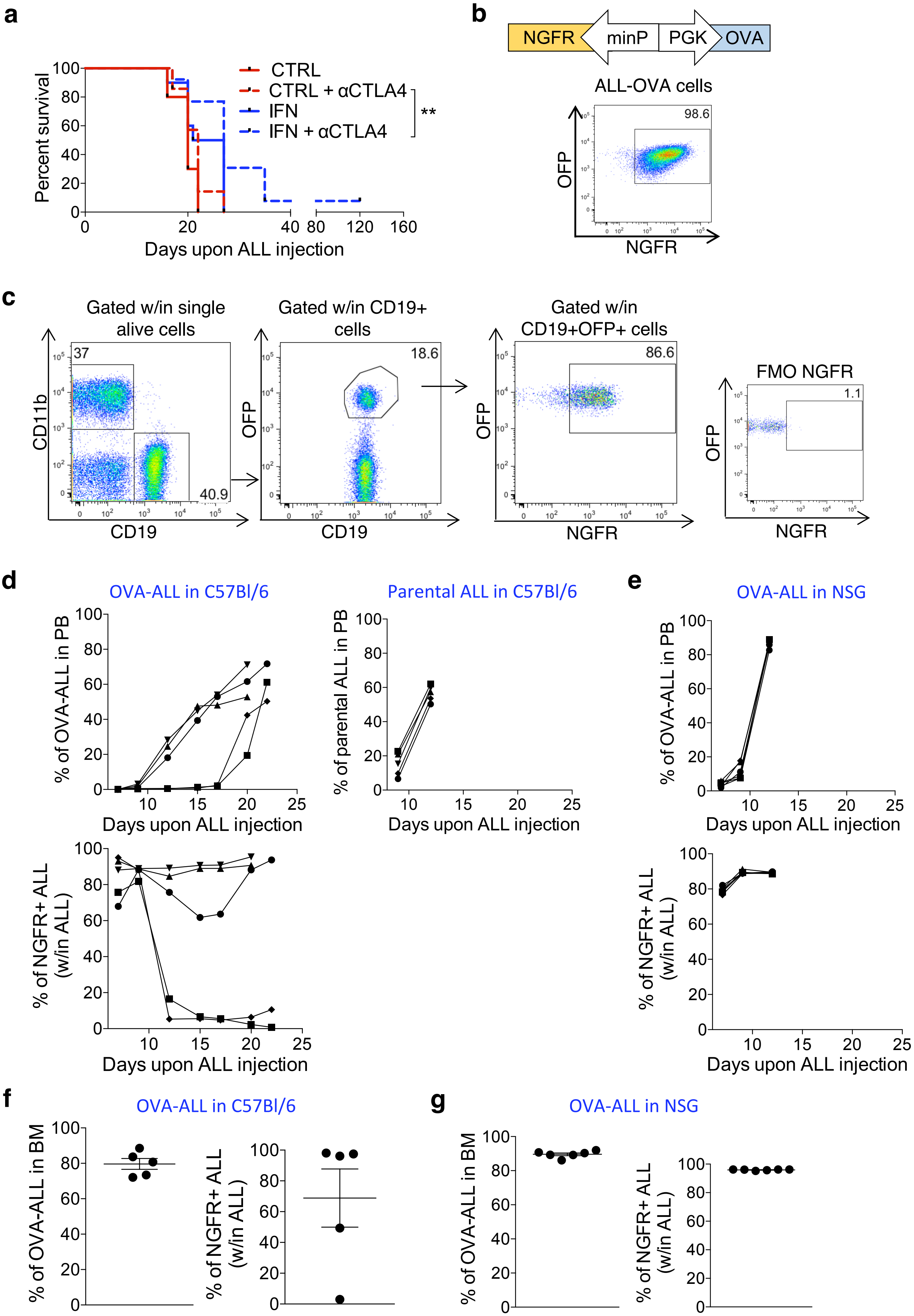

a
Percent survival
CTRL
CTRL + αCTLA4
IFN
IFN + αCTLA4
**
Days upon ALL injection
b
NGFR
minP
PGK
OVA
ALL-OVA cells
98.6
OFP
NGFR
c
Gated w/in single alive cells
37
40.9
CD11b
CD19
Gated w/in CD19+ cells
18.6
OFP
CD19
Gated w/in CD19+OFP+ cells
86.6
OFP
NGFR
FMO NGFR
1.1
OFP
NGFR
d
OVA-ALL in C57Bl/6
% of OVA-ALL in PB
Days upon ALL injection
Parental ALL in C57Bl/6
% of parental ALL in PB
Days upon ALL injection
% of NGFR+ ALL (w/in ALL)
Days upon ALL injection
e
OVA-ALL in NSG
% of OVA-ALL in PB
Days upon ALL injection
% of NGFR+ ALL (w/in ALL)
Days upon ALL injection
f
OVA-ALL in C57Bl/6
% of OVA-ALL in BM
% of NGFR+ ALL (w/in ALL)
g
OVA-ALL in NSG
% of OVA-ALL in BM
% of NGFR+ ALL (w/in ALL)

Supplementary Figure 1. Engineered OVA-ALL showed increased immunogenicity as compared to parental ALL. **a**, Survival curve of ALL-injected CTRL (n=10, treated with isotype control antibody), IFN (n=10, treated with isotype control antibody), CTRL + αCTLA4 (n=14) and IFN + αCTLA4 (n=13) mice. **p<0.01, Mantel-Haenszel test. **b**, Schematic representation of the bidirectional LV (BdLV) used to generate OVA-ALL with representative plot showing NGFR expression on purified OVA-ALL. **c**, Gating strategy used to identify ALL (CD19+OFP+) and NGFR expression on ALL in PB of tumor-injected mice. **d**, **e**, Top: Percentage (mean ± SEM) of OVA-ALL in the PB of immune-competent C57Bl/6 (n=5) (**d**) and immune-deficient NSG (n=6) (**e**) mice and of parental ALL in the PB of immune-competent C57Bl/6 (n=5) (**d**) mice. Bottom: expression of the NGFR marker on ALL present in the PB of immune-competent C57Bl/6 (n=5) (**d**) and immune-deficient NSG (n=6) (**e**) mice. Note that expression of NGFR and OVA antigens is co-regulated by a bidirectional promoter present within the lentiviral vector. **f**, **g**, Percentage (mean ± SEM) of OVA-ALL and expression of NGFR marker on leukemic cells present in the BM of immune-competent C57Bl/6 (n=6) (**f**) and immune-deficient NSG (n=6) (**g**) mice at the time of sacrifice.

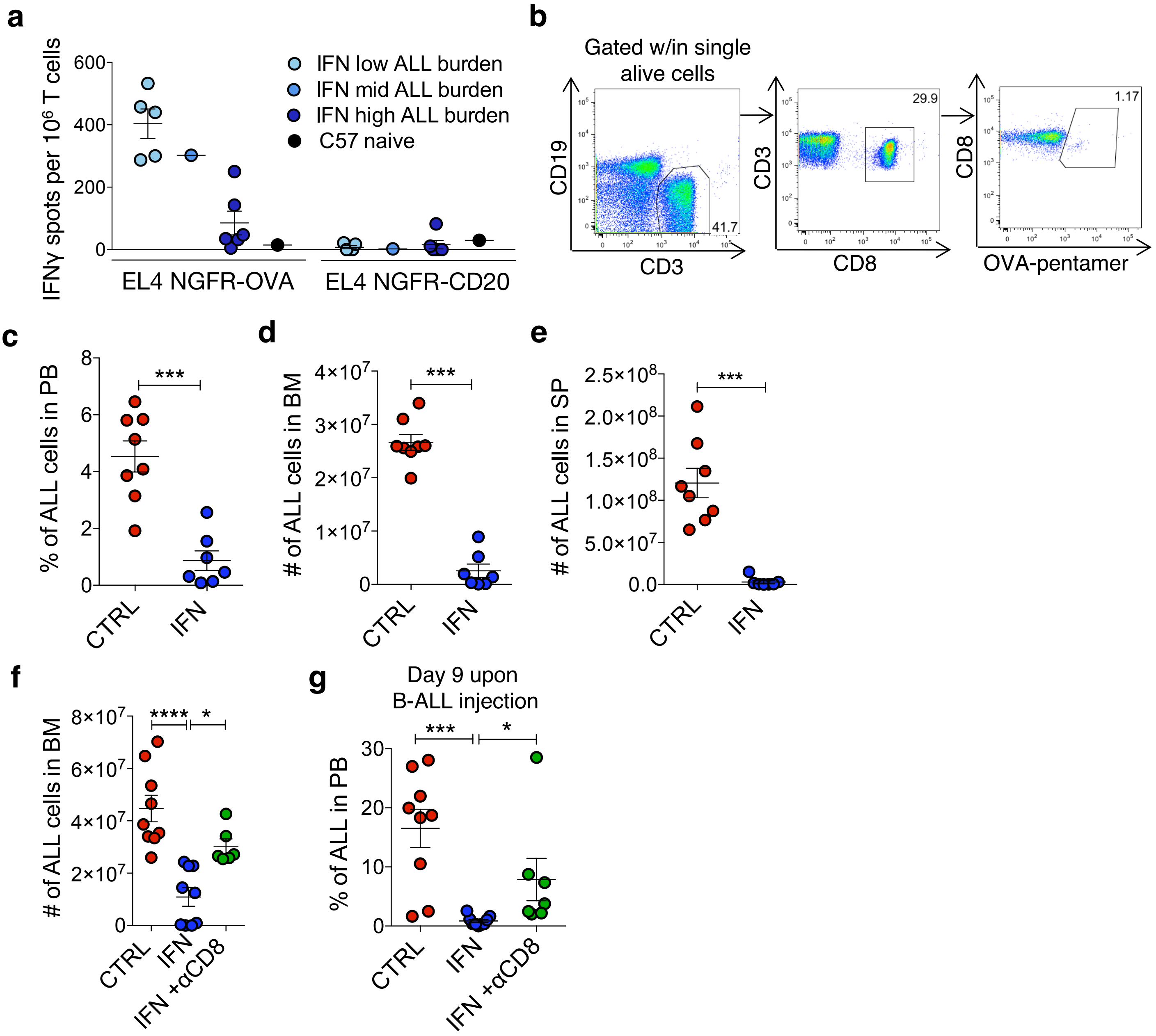
a
IFNγ spots per 10^6 T cells
IFN low ALL burden
IFN mid ALL burden
IFN high ALL burden
C57 naive
EL4 NGFR-OVA
EL4 NGFR-CD20
b
Gated w/in single alive cells
CD19
CD3
41.7
CD3
CD8
29.9
CD8
OVA-pentamer
1.17
c
% of ALL cells in PB
CTRL
IFN
d
# of ALL cells in BM
CTRL
IFN
e
# of ALL cells in SP
CTRL
IFN
f
# of ALL cells in BM
CTRL
IFN
IFN +αCD8
g
Day 9 upon B-ALL injection
% of ALL in PB
CTRL
IFN
IFN +αCD8

Supplementary Figure 2. ALL growth inhibition and induction of OVA-specific T cells in IFN mice. a, Splenic CD8+ T cells from IFN mice, 13 days upon ALL-OVA injection, or from non tumor-bearing CTRL (naïve C57Bl/6, n=1) mice tested by IFNγ-ELISPOT against the EL4 target cell line transduced with NGFR-OVA or NGFR-hCD20 bidirectional lentiviral vector. Each dot represents a mouse, mean ± SEM. Low tumor burden (n=5): 5.2 ± 4.9 (mean ± SEM) % OVA-ALL; mid tumor burden (n=1): 33% OVA-ALL; high tumor burden (n=6): 51 ± 4.9 % OVA-ALL. **b**, Gating strategy used to identify OVA-specific CD8+ T cells in the PB of the mice. **c**, **d**, **e**, Percentage (**c**) and absolute numbers (**d**, **e**) (mean ± SEM) of OVA-ALL in PB (**c**) and in BM and spleen (**d**, **e**) of IFN (n=14-6) and CTRL (n=15-8) mice, 9 days upon tumor injection. ***P<0.001, Mann-Whitney. Each dot represents a mouse. **f**, **g**, Absolute numbers and percentage of OVA-ALL in the BM (**f**, 16 days upon tumor challenge) and PB (**g**, 9 days upon tumor challenge) of IFN (n=9), CD8-depleted IFN (n=7) and CTRL (n=9) mice. Each dot represents a mouse. *p<0.05, ***p<0.001, ****p<0.0001, Kruskall-Wallis with adjusted by Dunn's test.

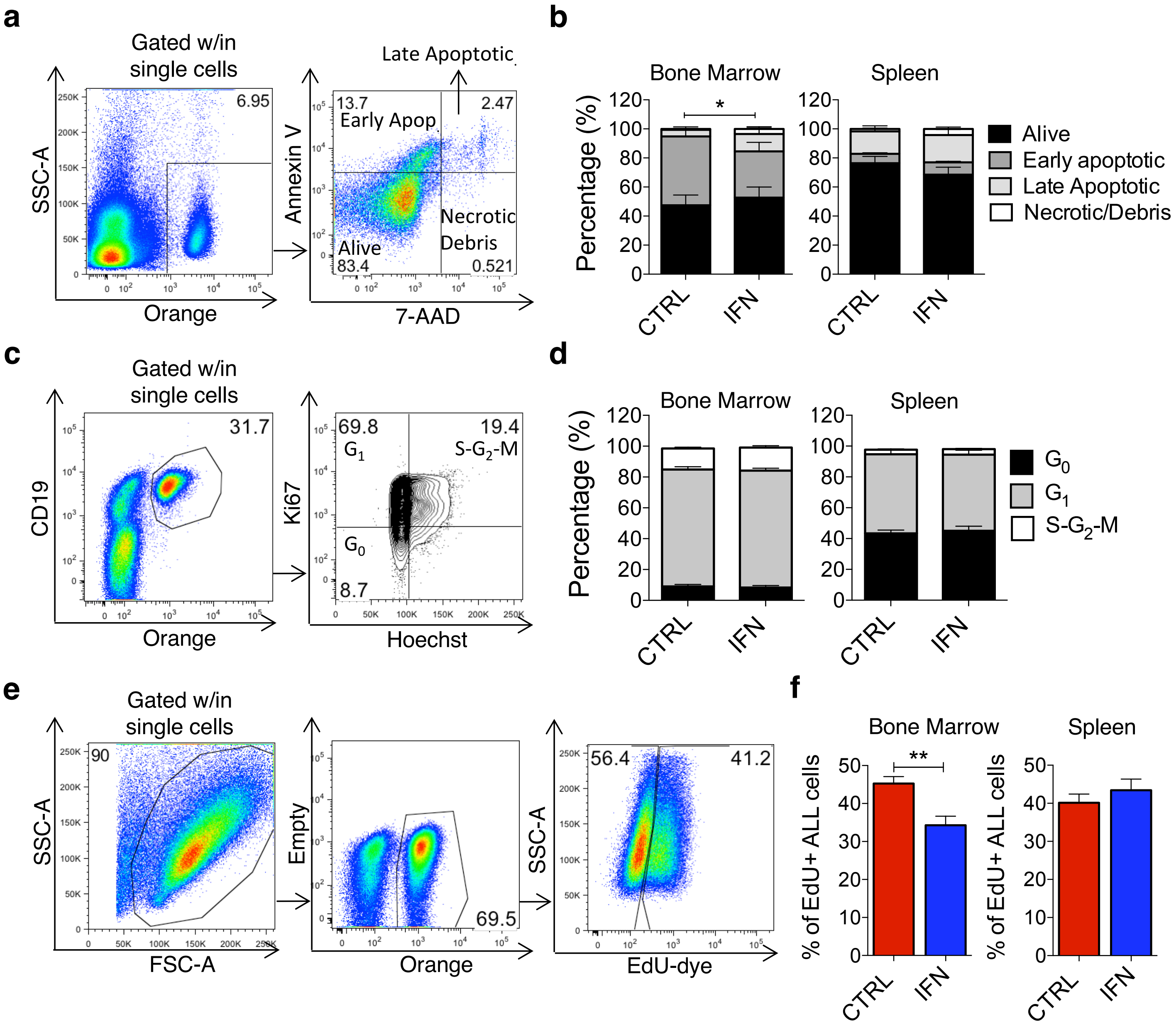
a
Gated w/in single cells
SSC-A
Orange
6.95
Annexin V
7-AAD
Late Apoptotic
13.7
Early Apop
2.47
Necrotic Debris
Alive
83.4
0.521
b
Bone Marrow
Spleen
Percentage (%)
Alive
Early apoptotic
Late Apoptotic
Necrotic/Debris
CTRL
IFN
c
Gated w/in single cells
CD19
Orange
31.7
Ki67
Hoechst
69.8
G1
19.4
S-G2-M
G0
8.7
d
Bone Marrow
Spleen
Percentage (%)
G0
G1
S-G2-M
CTRL
IFN
e
Gated w/in single cells
SSC-A
FSC-A
90
Empty
Orange
69.5
SSC-A
EdU-dye
56.4
41.2
f
Bone Marrow
Spleen
% of EdU+ ALL cells
CTRL
IFN

Supplementary Figure 3. Gating strategy used to assess apoptosis, cell cycle distribution and proliferation rate of ALL cells. **a**, **b**, Gating strategy (**a**) and analysis (**b**) (percentage, mean ± SEM) of early apoptotic (Annexin+7-AAD-), late apoptotic (Annexin+7-ADD+), dead/necrotic (Annexin-7AAD+) and alive (Annexin-7-AAD-) OVA-ALL cells present in BM and spleen of IFN (n=11) and CTRL (n=15) mice, 9 days upon tumor injection. *p<0.05, Mann-Whitney. **c**, **d**, Gating strategy (**c**) and analysis (**d**: percentage, mean ± SEM) of OVA-ALL distribution in the G_0 (Ki67-Hoechst^{low}), G_1 (Ki67+Hoechst^{low}) and S-G_2-M (Ki67+Hoechst^{high}) phase of the cell cycle in BM and spleen of IFN (n=11) and CTRL (n=15) mice, 9 days upon tumor injection. **e**, **f**, Gating strategy (**e**) and analysis (**f**: percentage, mean ± SEM) of proliferating EdU+ ALL cells present in BM and spleen of IFN (n=11) and CTRL (n=15) mice, 9 days upon OVA-ALL injection. **p<0.01, Mann-Whitney.

a

b

c

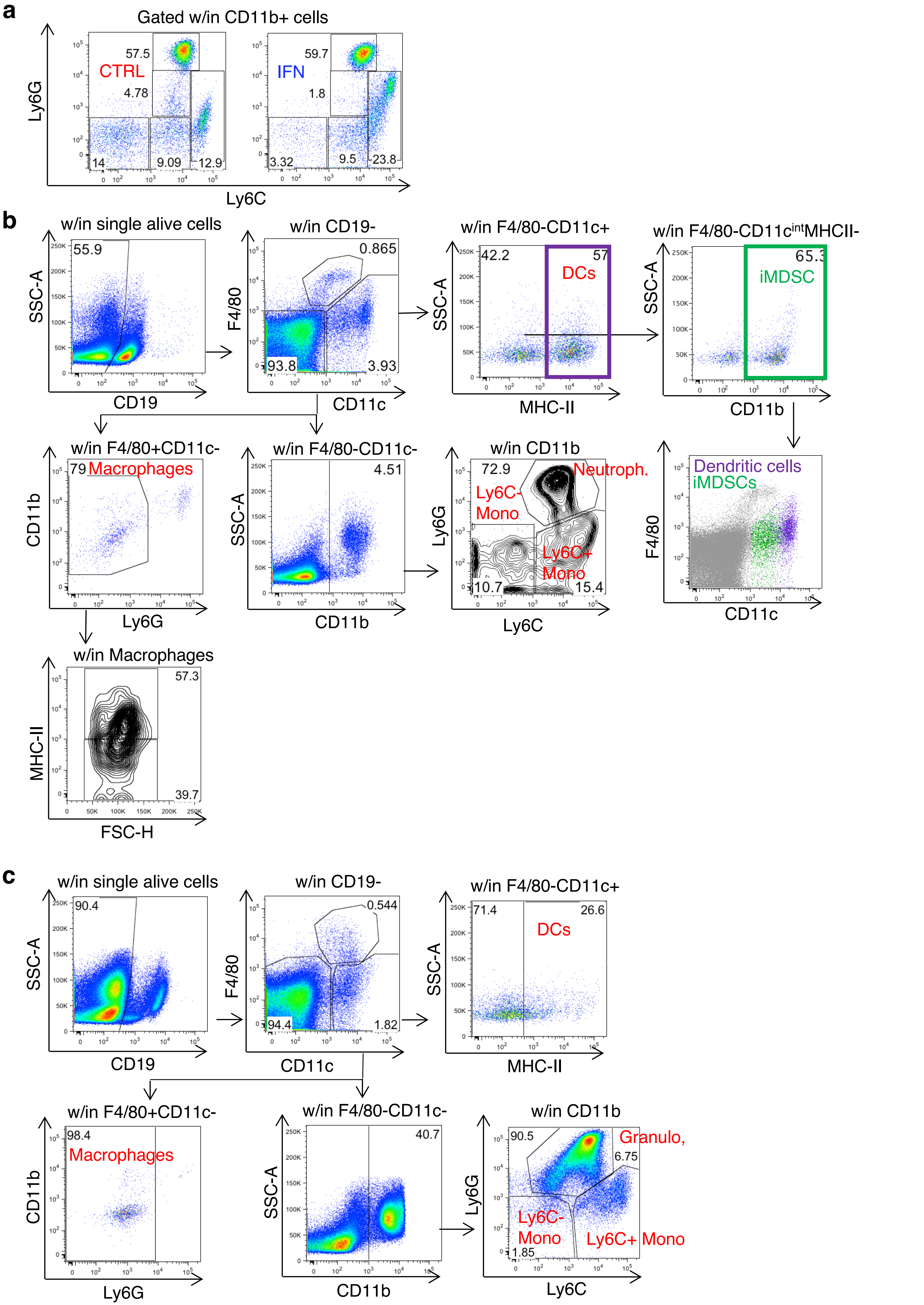

Gated w/in CD11b+ cells
CTRL
IFN
57.5
4.78
14
9.09
12.9
59.7
1.8
3.32
9.5
23.8
Ly6G
Ly6C
w/in single alive cells
SSC-A
CD19
55.9
w/in CD19-
F4/80
CD11c
0.865
93.8
3.93
w/in F4/80-CD11c+
SSC-A
MHC-II
42.2
57
DCs
w/in F4/80-CD11c^int MHCII-
SSC-A
CD11b
65.3
iMDSC
w/in F4/80+CD11c-
79
Macrophages
CD11b
Ly6G
w/in F4/80-CD11c-
4.51
SSC-A
CD11b
w/in CD11b
72.9
Neutroph.
Ly6C-
Mono
Ly6C+
Mono
10.7
15.4
Ly6G
Ly6C
Dendritic cells
iMDSCs
F4/80
CD11c
w/in Macrophages
57.3
39.7
MHC-II
FSC-H
w/in single alive cells
90.4
SSC-A
CD19
w/in CD19-
0.544
94.4
1.82
F4/80
CD11c
w/in F4/80-CD11c+
71.4
26.6
DCs
SSC-A
MHC-II
w/in F4/80+CD11c-
98.4
Macrophages
CD11b
Ly6G
w/in F4/80-CD11c-
40.7
SSC-A
CD11b
w/in CD11b
90.5
Granulo,
6.75
Ly6C-
Mono
Ly6C+ Mono
1.85
Ly6G
Ly6C

Supplementary Figure 4. Flow cytometric analysis of immune cells in the peripheral blood, spleen and BM of tumor-bearing and non tumor-injected IFN and CTRL mice. a, Representative plots showing the distribution of neutrophils (Ly6G+Ly6Cint), classical monocytes (LyC+Ly6Gint), non-classical monocytes (Ly6C-Ly6G-) and Ly6CintLy6G- cells within total myeloid cells in the PB of CTRL and IFN mice. **b**, **c**, Gating strategy of the indicated populations in the spleen (**b**) and BM (**c**) of tumor-free and OVA-ALL-injected IFN and CTRL mice (CTRL: n=3, IFN n=2, CTRL + ALL: n=9, IFN + ALL: n=7), 10 days upon tumor injection. Macrophages (MΦ) are identified as CD19-CD11c-CD11b+Ly6G-F4/80+ cells. Splenic macrophages are further distinguished into MHC-II+ and MHC-II- MΦ using the IAb (MHC-II) marker. We distinguished CD19-F4/80-CD11c+MHCII-CD11b+ immature myeloid derived suppressor cells (iMDSCs) from the CD19-F4/80-CD11c+MHCII+ dendritic cells (DC). We defined granulocytes as CD19-F4/80-CD11c-CD11b+Ly6CintLy6G+, classical monocytes as CD19-F4/80-CD11c-CD11b+Ly6C+Ly6G- and non classical monocytes as CD19-F4/80-CD11c-CD11b+Ly6C-Ly6G-.

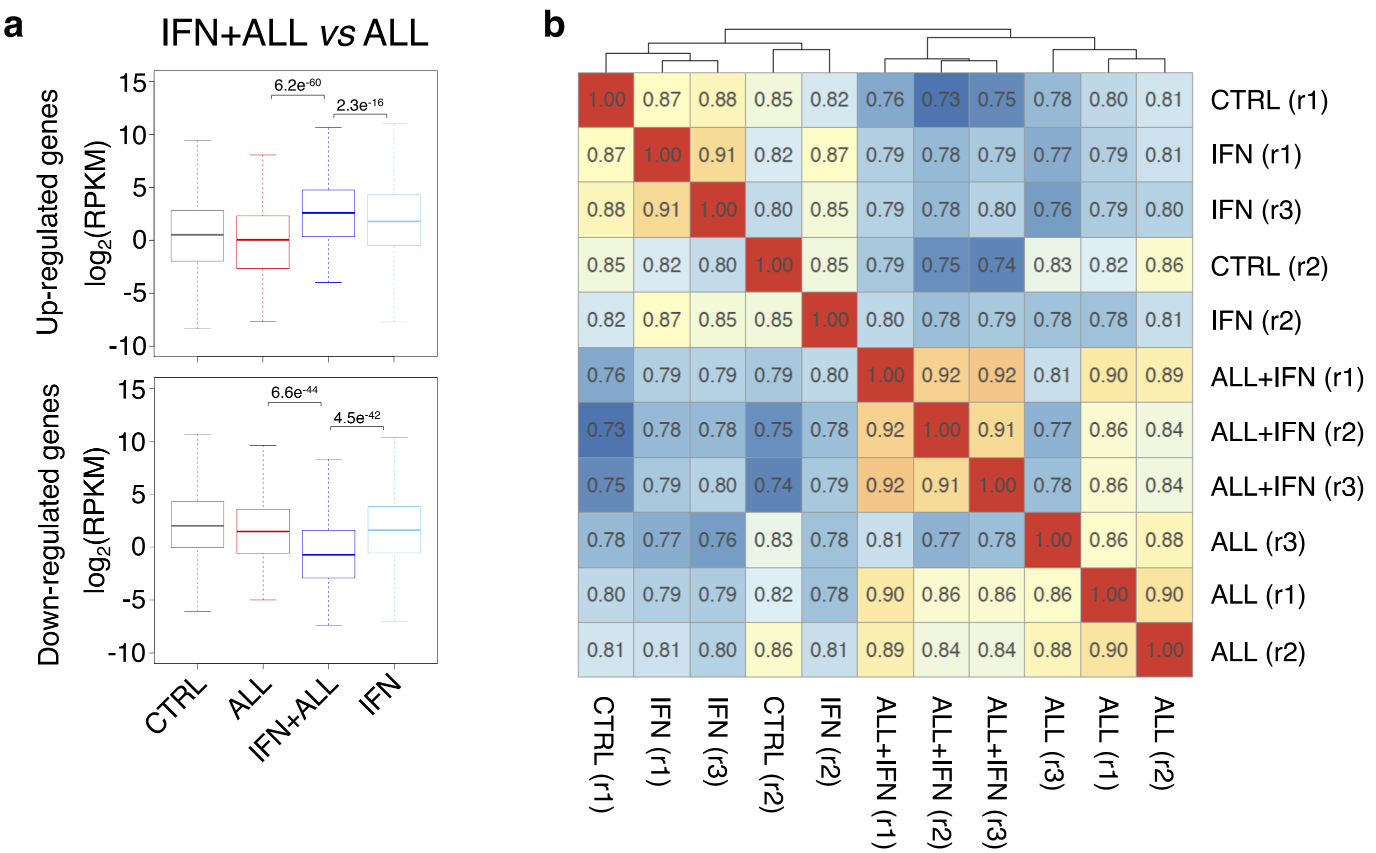
a
IFN+ALL vs ALL
Up-regulated genes
log2(RPKM)
15
10
5
0
-5
-10
6.2e-60
2.3e-16
Down-regulated genes
log2(RPKM)
6.6e-44
4.5e-42
CTRL
ALL
IFN+ALL
IFN
b
CTRL (r1) 1.00 0.87 0.88 0.85 0.82 0.76 0.73 0.75 0.78 0.80 0.81
IFN (r1) 0.87 1.00 0.91 0.82 0.87 0.79 0.78 0.79 0.77 0.79 0.81
IFN (r3) 0.88 0.91 1.00 0.80 0.85 0.79 0.78 0.80 0.76 0.79 0.80
CTRL (r2) 0.85 0.82 0.80 1.00 0.85 0.79 0.75 0.74 0.83 0.82 0.86
IFN (r2) 0.82 0.87 0.85 0.85 1.00 0.80 0.78 0.79 0.78 0.78 0.81
ALL+IFN (r1) 0.76 0.79 0.79 0.79 0.80 1.00 0.92 0.92 0.81 0.90 0.89
ALL+IFN (r2) 0.73 0.78 0.78 0.75 0.78 0.92 1.00 0.91 0.77 0.86 0.84
ALL+IFN (r3) 0.75 0.79 0.80 0.74 0.79 0.92 0.91 1.00 0.78 0.86 0.84
ALL (r3) 0.78 0.77 0.76 0.83 0.78 0.81 0.77 0.78 1.00 0.86 0.88
ALL (r1) 0.80 0.79 0.79 0.82 0.78 0.90 0.86 0.86 0.86 1.00 0.90
ALL (r2) 0.81 0.81 0.80 0.86 0.81 0.89 0.84 0.84 0.88 0.90 1.00
CTRL (r1)
IFN (r1)
IFN (r3)
CTRL (r2)
IFN (r2)
ALL+IFN (r1)
ALL+IFN (r2)
ALL+IFN (r3)
ALL (r3)
ALL (r1)
ALL (r2)

Supplementary Figure 5. Bulk RNA-Seq analyses in splenic macrophages. a, Box plots showing expression levels, in the indicated conditions, of up-regulated (n=213) or down-regulated (n=258) genes in macrophages from ALL mice treated with IFN gene therapy (IFN+ALL) as compared to ALL controls. Numbers represent results of Wilcoxon signed-rank test in the indicated comparisons. **b**, Heatmap of correlation between RNA-Seq datasets in macrophages from the indicated conditions. Numbers indicate calculated coefficients of determination (R^2). Samples are ordered based on unsupervised hierarchical clustering.

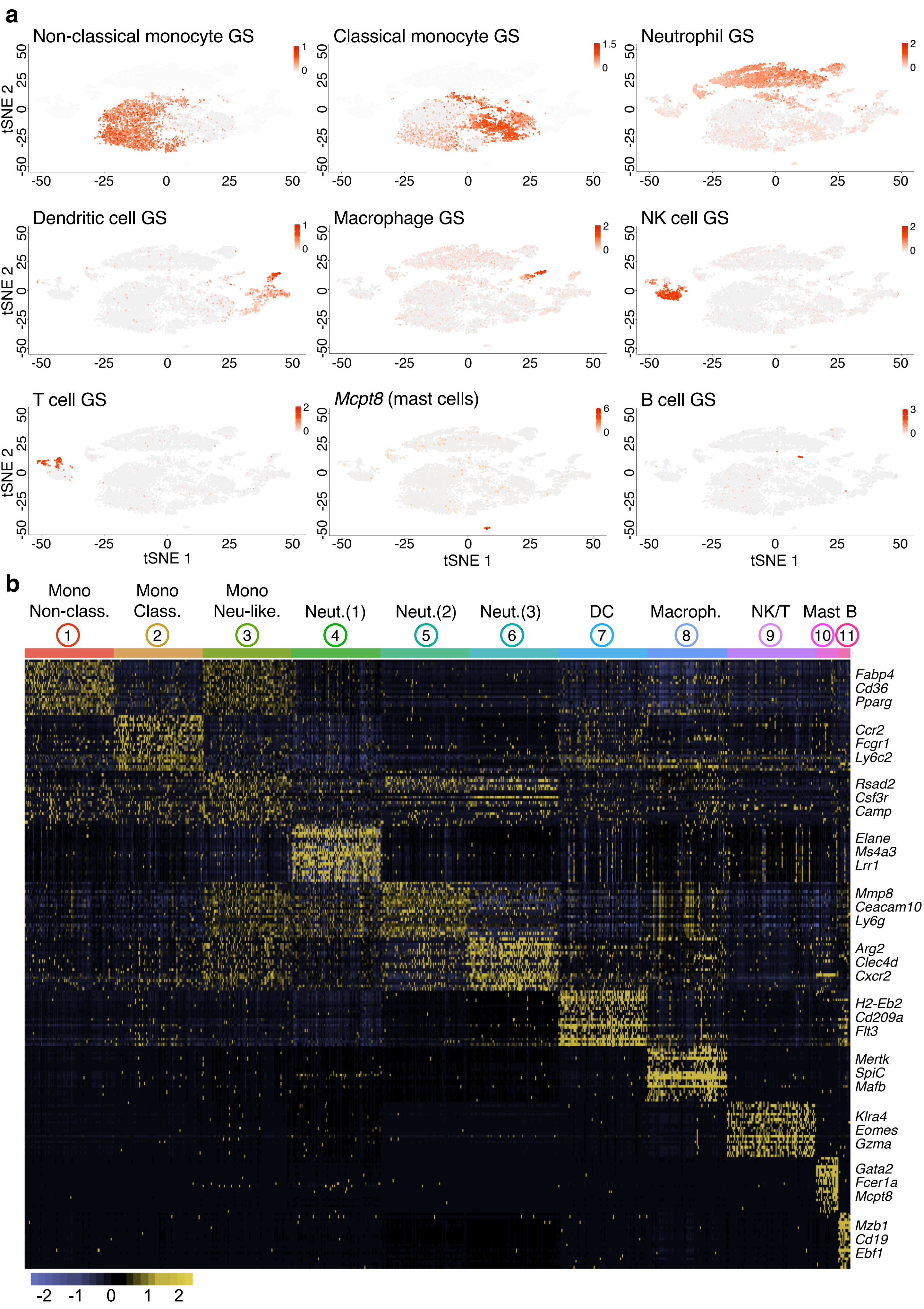

a
Non-classical monocyte GS
Classical monocyte GS
Neutrophil GS
Dendritic cell GS
Macrophage GS
NK cell GS
T cell GS
Mcpt8 (mast cells)
B cell GS
tSNE 2
tSNE 1
b
Mono Non-class.
Mono Class.
Mono Neu-like.
Neut.(1)
Neut.(2)
Neut.(3)
DC
Macroph.
NK/T
Mast
B
1 2 3 4 5 6 7 8 9 10 11
Fabp4
Cd36
Pparg
Ccr2
Fcgr1
Ly6c2
Rsad2
Csf3r
Camp
Elane
Ms4a3
Lrr1
Mmp8
Ceacam10
Ly6g
Arg2
Clec4d
Cxcr2
H2-Eb2
Cd209a
Flt3
Mertk
SpiC
Mafb
Klra4
Eomes
Gzma
Gata2
Fcer1a
Mcpt8
Mzb1
Cd19
Ebf1
-2 -1 0 1 2

Supplementary Figure 6. Cell type identification in scRNA-Seq data from splenic CD11b$^+$ cells. a, tSNE plots showing single-cell expression levels of the indicated gene signatures (GS). Non-classical monocytes: Cd300e, 1700011I03Rik, Tspan9, Dmpk, Fxyd2; classical monocytes: Ly6c2, Tarm, Tfec, Psrc1, Mmp8; neutrophils: Il1f9, Pglyrp4, Nlrp12, Mrgpra2b, Mmp8, Ltf, Amer2, Stfa2l1, Gm5483; dendritic cells: Adam23, Procr, Mab21l3, Sucnr1, Fndc5, Rnf186, Flt3, Cd207, Adam11, Apol7c, Htr7; macrophages: Vcam1, Mertk, Actn1, Fcna, Crip2, Spic, Kcna2, Gfra2, Stab2, Jup; NK cells: Khdc1c, Khdc1b, Phactr3, Col8a2, Klra4, Adamts14, Gzma, Cma1, Gzmb, Clip4; T cells: Cd3g, Cd3e, Bcl11b, Actn2, Camk4; B cells: Pax5, Cd79a, Cd19, Chst3, Scn4a, Cacna1i, Ly6d, Klhl14, Mzb1. Colour scale reflects average expression (log transformed TPM) across genes within each signature. **b**, Heatmap showing expression (scaled log transformed TPM values) of top 20 discriminative genes for each cluster. Selected representative genes for each cluster are shown on the right. Up to 200 single cells are shown for each cluster.

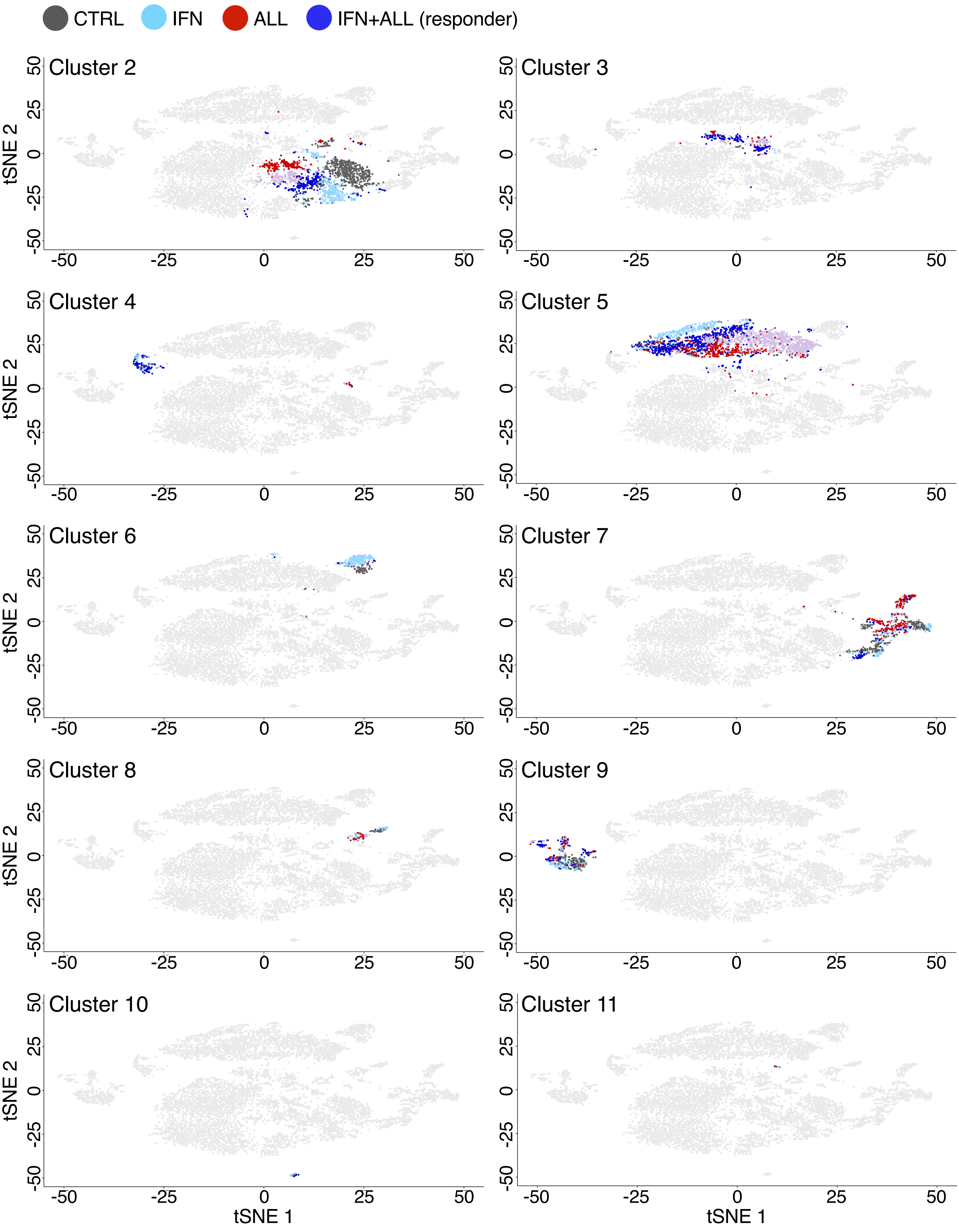

CTRL
IFN
ALL
IFN+ALL (responder)
Cluster 2
Cluster 3
Cluster 4
Cluster 5
Cluster 6
Cluster 7
Cluster 8
Cluster 9
Cluster 10
Cluster 11
tSNE 1
tSNE 2

Supplementary Figure 7. Leukemia and IFN-induced changes in scRNA-Seq data on splenic CD11b$^+$ cells. tSNE plots showing scRNA-Seq data from clusters 2-11, coloured based on the experimental condition.

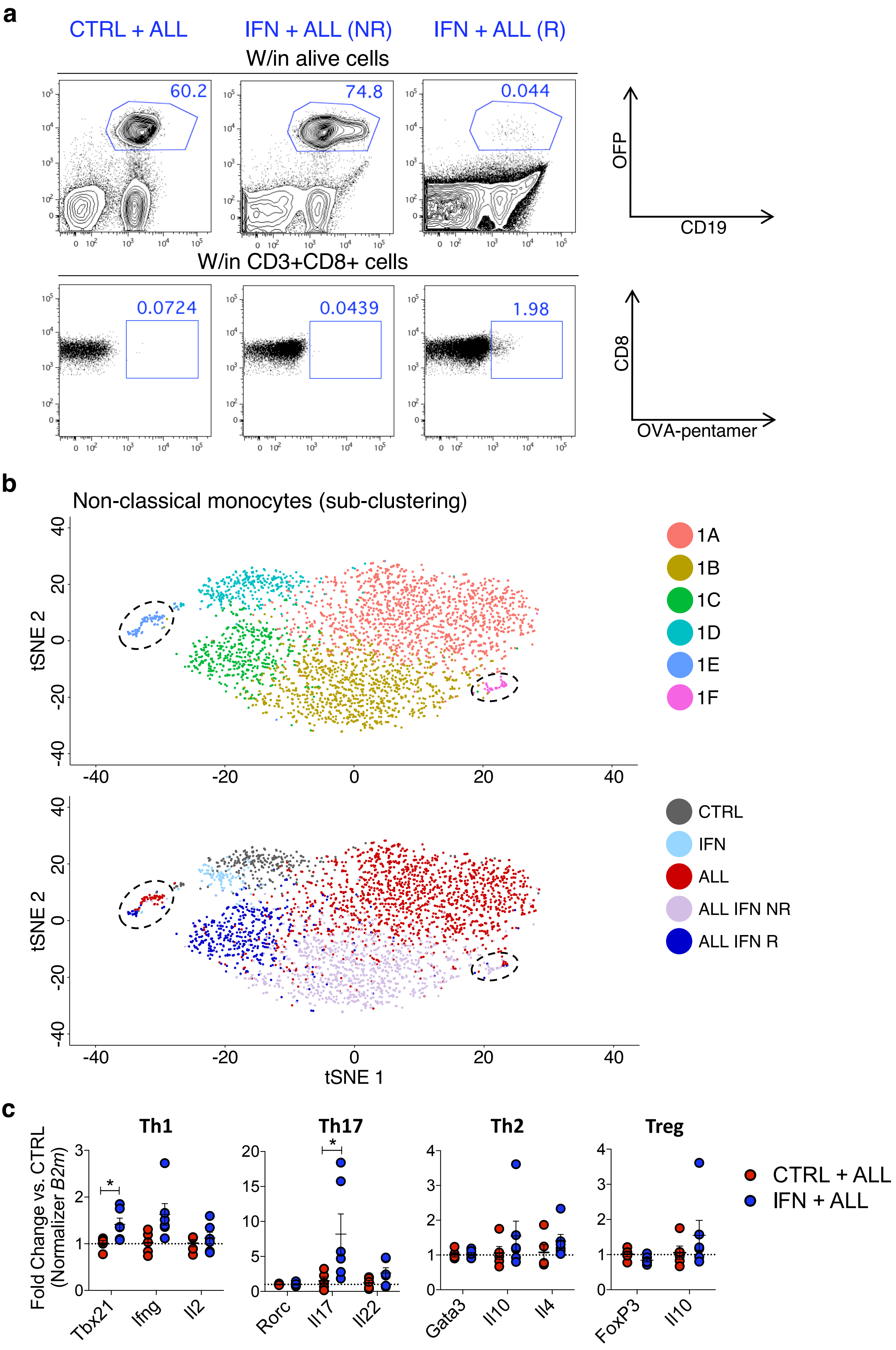
a
CTRL + ALL
IFN + ALL (NR)
IFN + ALL (R)
W/in alive cells
60.2
74.8
0.044
OFP
CD19
W/in CD3+CD8+ cells
0.0724
0.0439
1.98
CD8
OVA-pentamer
b
Non-classical monocytes (sub-clustering)
tSNE 2
tSNE 1
1A
1B
1C
1D
1E
1F
CTRL
IFN
ALL
ALL IFN NR
ALL IFN R
c
Th1
Th17
Th2
Treg
Fold Change vs. CTRL (Normalizer B2m)
Tbx21
Ifng
Il2
Rorc
Il17
Il22
Gata3
Il10
Il4
FoxP3
Il10
CTRL + ALL
IFN + ALL

Supplementary Figure 8. Sub-clustering analysis of scRNA-Seq data from non-classical monocytes and gene expression analyses on CD4 T cells. a, Flow cytometric plots showing OVA-ALL cells (top) and OVA-specific CD8 T cells (bottom) present in the spleen from representative ALL-injected CTRL (CTRL + ALL), IFN non responder (IFN + ALL NR) and IFN responder (IFN + ALL R) mice. CD11b+ cells from these mice were analysed by scRNA-seq. **b**, tSNE plots showing sub-cluster of cells within the non-classical monocyte population, coloured based on clustering (top plot) or experimental condition (bottom plot). Circled sub-clusters represent minor sub-clusters with dispersed composition and correspond to those marked by asterisks in Fig. 3i. **c**, Expression analysis by RT-qPCR of the indicated Th1, Th17, Th2 and Treg genes (mean fold-change over OVA-ALL-injected CTRL mice ± SEM) in splenic CD4+ T cells purified from OVA-ALL-injected IFN (n=6) and CTRL (n=5) mice, 9 days upon tumor injection. Each dot represents a mouse. $^{*}p<0.05$, Mann-Whitney.

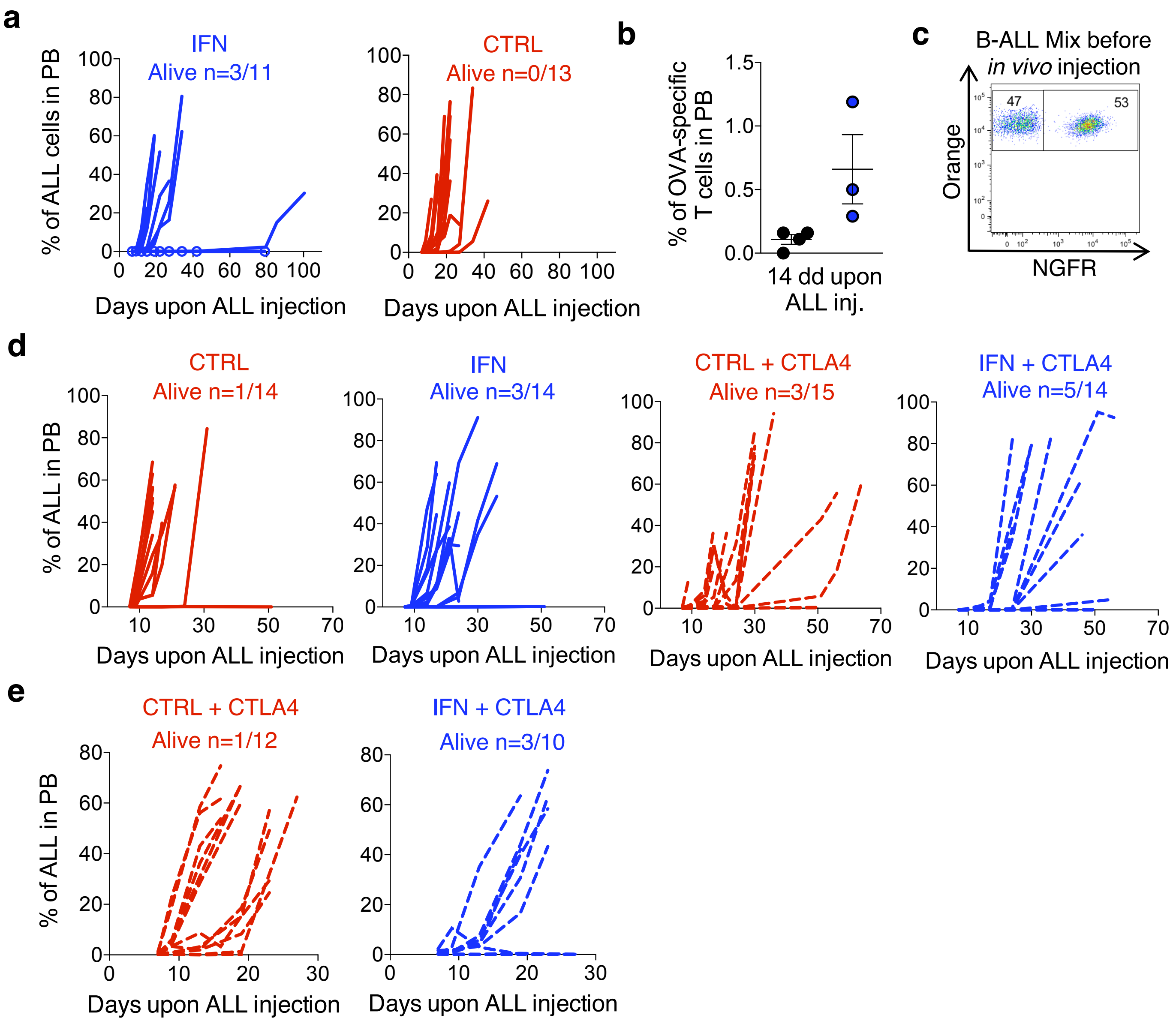
a
IFN
Alive n=3/11
% of ALL cells in PB
Days upon ALL injection
CTRL
Alive n=0/13
Days upon ALL injection
b
% of OVA-specific T cells in PB
14 dd upon ALL inj.
c
B-ALL Mix before in vivo injection
47
53
Orange
NGFR
d
CTRL
Alive n=1/14
% of ALL in PB
Days upon ALL injection
IFN
Alive n=3/14
Days upon ALL injection
CTRL + CTLA4
Alive n=3/15
Days upon ALL injection
IFN + CTLA4
Alive n=5/14
Days upon ALL injection
e
CTRL + CTLA4
Alive n=1/12
% of ALL in PB
Days upon ALL injection
IFN + CTLA4
Alive n=3/10
Days upon ALL injection

Supplementary Figure 9. ALL growth kinetic in individual mice. a, Percentage (mean ± SEM) of OVA-ALL in the PB of each individual IFN (n=11) and CTRL (n=13) mouse. Each line represents a mouse. **b**, Percentage (mean ± SEM) of OVA-specific T cells in surviving IFN mice (n=3, from Fig. 4a) vs. 4 naïve mice, 14 days upon second tumor challenge with OVA-ALL. Each dot represents a mouse. **c**, Representative flow cytometric plot showing the OVA-ALL and parental ALL cells mixed at 1 to 1 ratio before injection in the mice. **d**, **e**, Percentage (mean ± SEM) of OVA-ALL in the PB of each individual (**d**) IFN (n=14), CTRL (n=14), IFN + αCTLA4 (n=14) and CTRL + αCTLA4 (n=15) mouse and (**e**) IFN + αCTLA4 (n=10) and CTRL + αCTLA4 (n=12) mouse. Each line represents a mouse.

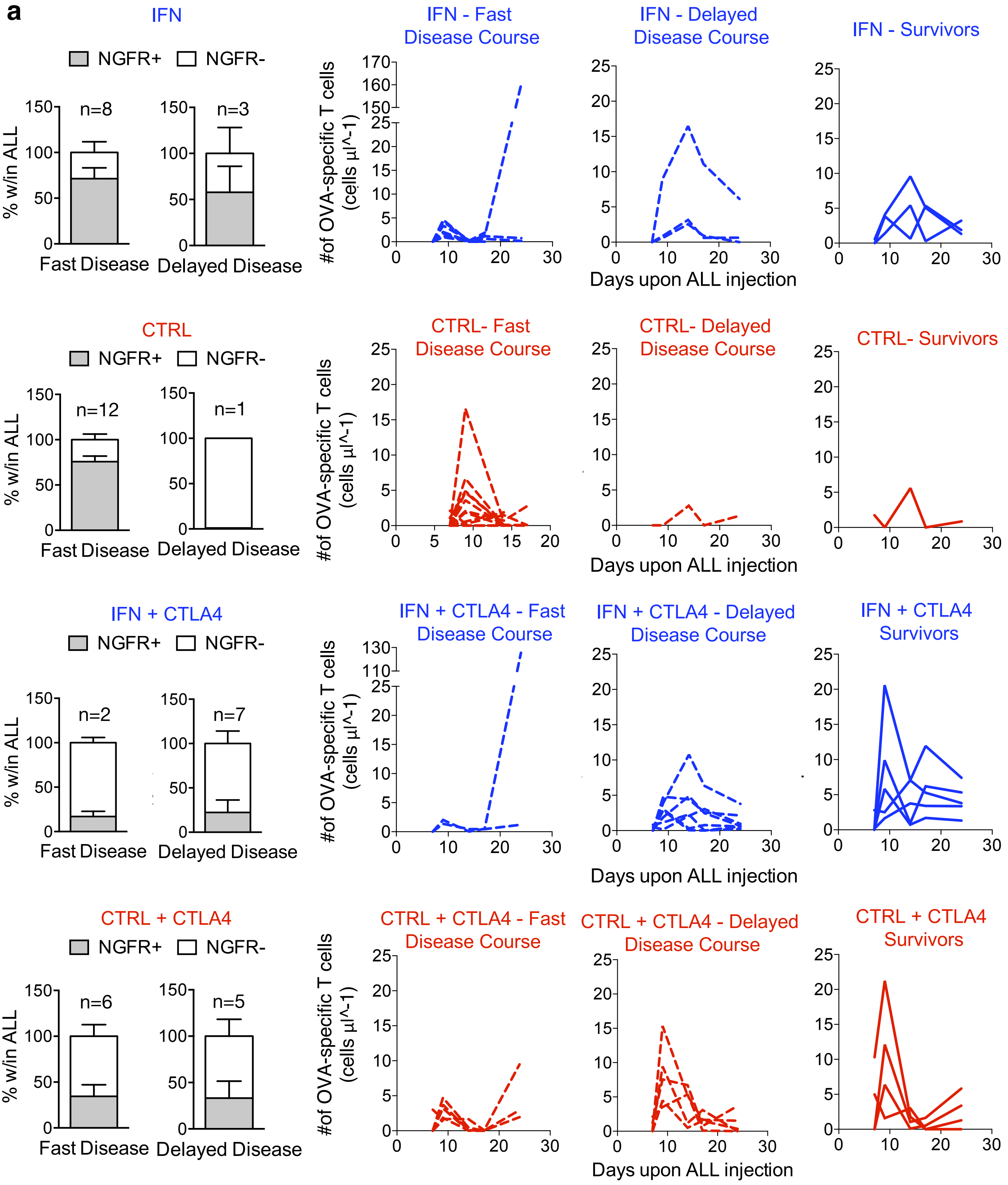
a
IFN
NGFR+ NGFR-
% w/in ALL
n=8
n=3
Fast Disease
Delayed Disease
IFN - Fast Disease Course
IFN - Delayed Disease Course
IFN - Survivors
#of OVA-specific T cells (cells µl^-1)
Days upon ALL injection
CTRL
n=12
n=1
CTRL- Fast Disease Course
CTRL- Delayed Disease Course
CTRL- Survivors
IFN + CTLA4
n=2
n=7
IFN + CTLA4 - Fast Disease Course
IFN + CTLA4 - Delayed Disease Course
IFN + CTLA4 Survivors
CTRL + CTLA4
n=6
n=5
CTRL + CTLA4 - Fast Disease Course
CTRL + CTLA4 - Delayed Disease Course
CTRL + CTLA4 Survivors

Supplementary Figure 10. Immune-selection of OVA-negative ALL cells is enhanced in CTLA4-treated IFN and CTRL mice. a, NGFR expression on BM-infiltrating ALL (or PB ALL for those mice for which BM analysis is not available) and absolute numbers (mean ± SEM) of OVA-specific T cells (each line represents a mouse) in the PB of IFN (n=14), CTRL (n=14), IFN+αCTLA4 (n=14) and CTRL+αCTLA4 (n=15) mice showing fast, delayed disease course or long-term survival. Fast disease course: CTRL: 14-21 days (range), 15.66 ± 0.8 (mean ± SEM), n=12; IFN: 17-25 days, 21 ± 1.3, n=8; CTRL + CTLA4: 14-21 days, 19 ± 1.2, n=6; IFN + CTLA4: 25 days, 25 ± 0, n=2; delayed disease course: CTRL: 36 days, n=1; IFN: 30-36 days, 34 ± 2, n=3; CTRL + CTLA4, 30-64 days, 42 ± 7.4, n=5; IFN + CTLA4: 25-64 days, 44 ± 4.9, n=7). Surviving mice: CTRL: n=1; IFN: n=3, CTRL + CTLA4, n=4; IFN + CTLA4, n=5.

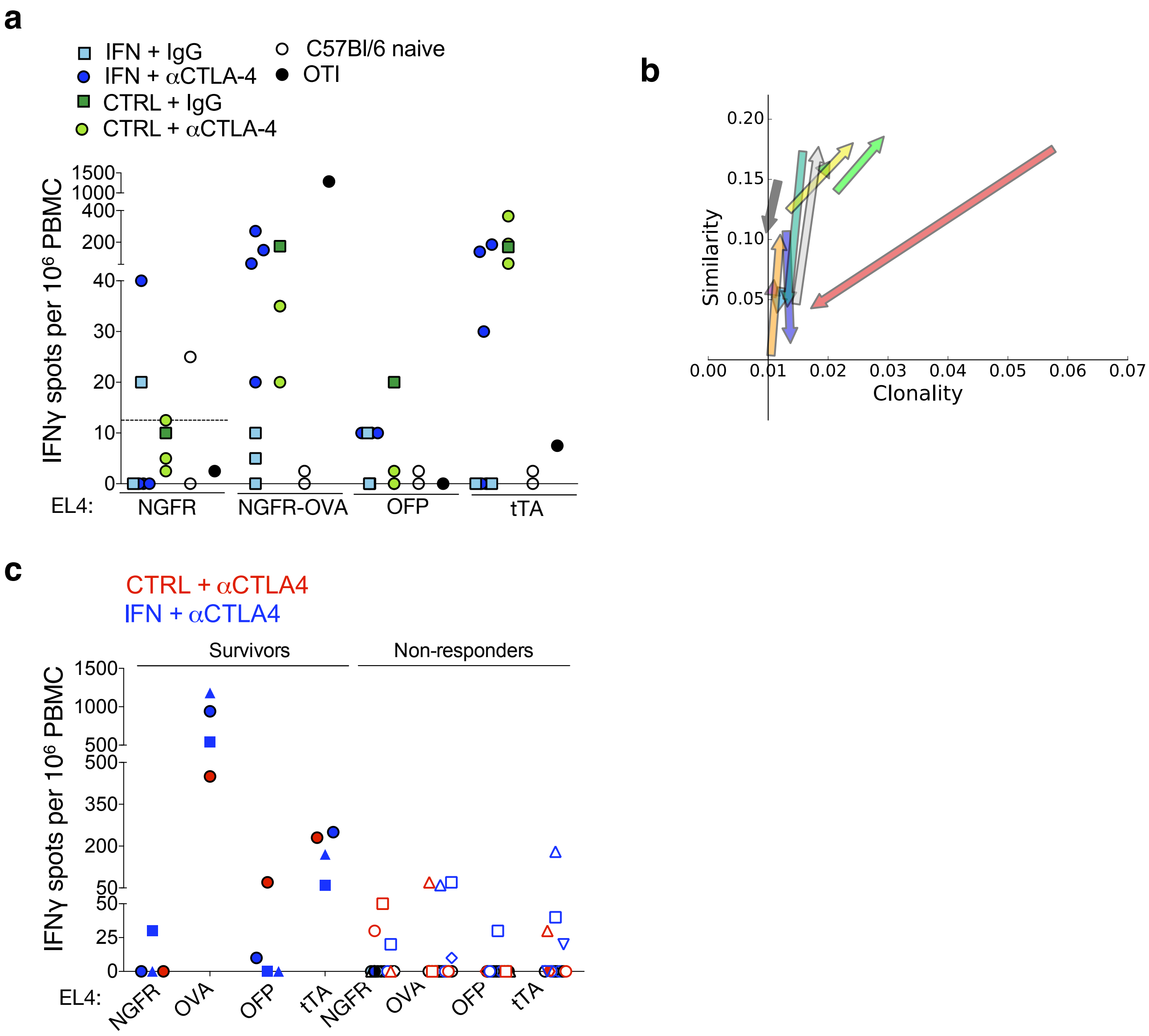
a
IFN + IgG
IFN + αCTLA-4
CTRL + IgG
CTRL + αCTLA-4
C57Bl/6 naive
OTI
IFNγ spots per 10^6 PBMC
1500
1000
400
200
40
30
20
10
0
EL4:
NGFR
NGFR-OVA
OFP
tTA
b
Similarity
0.20
0.15
0.10
0.05
0.00
0.01
0.02
0.03
0.04
0.05
0.06
0.07
Clonality
c
CTRL + αCTLA4
IFN + αCTLA4
Survivors
Non-responders
IFNγ spots per 10^6 PBMC
1500
1000
500
350
200
50
25
0
EL4:
NGFR
OVA
OFP
tTA
NGFR
OVA
OFP
tTA

Supplementary Figure 11. Combination of IFN gene therapy and CTLA blockade therapy promotes immune reactivity towards multiple tumor-specific surrogate antigens a, PBMC from long-term surviving mice from Fig. 5a (IFN n=3, CTRL n=1, IFN + αCTLA4 n=4, CTRL + αCTLA4 n=3) 51 days upon tumor injection and from non-tumor bearing (OT-I, n=1 and transplanted CTRL mice, n=2) mice tested by IFNγ-ELISPOT against the EL4 target cell line transduced with NGFR-OVA or NGFR-CD20 BdLV or PGK-OFP or PGK-tTA LV. The dashed line indicates the higher median background level seen against the EL4 NGFR-CD20 target cells. Each dot represents a mouse tested against all four tumor-specific surrogate antigens. **b**, Clonality and similarity of the TCR-beta CDR repertoire of tumor-free long-term surviving mice (each arrow represents a mouse) from Fig. 6b before OVA-ALL injection (start of the arrow, T_0) and at 30 days upon OVA-ALL challenge (tip of the arrow, T_1). **c**, PBMC from mice from Fig. 5d (IFN + αCTLA-4 survivors, full blue symbols, n=3; CTRL + αCTLA-4 survivors, full red symbols, n=1; IFN + αCTLA-4 non-responders, empty blue symbols, n=7; CTRL + αCTLA-4, empty red symbol, n=8 out of 12 non-responder controls) tested by IFNγ-ELISPOT as in (**a**), 19 days upon tumor injection. Each dot represents a mouse tested against all four tumor-specific surrogate antigens.

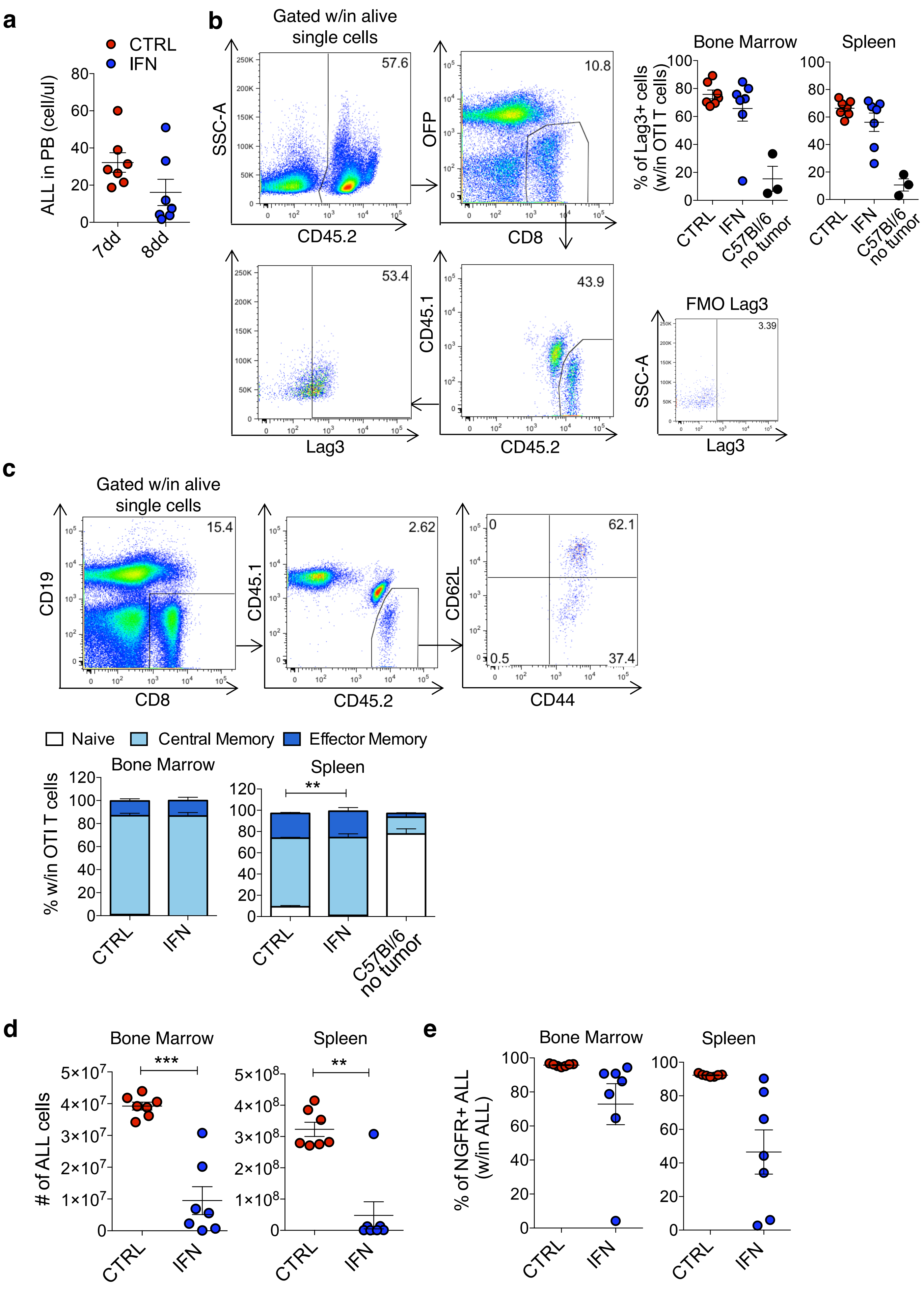

a
CTRL
IFN
ALL in PB (cell/ul)
7dd
8dd
b
Gated w/in alive single cells
SSC-A
CD45.2
57.6
OFP
CD8
10.8
Bone Marrow
Spleen
% of Lag3+ cells (w/in OTI T cells)
CTRL
IFN
C57Bl/6 no tumor
53.4
Lag3
CD45.1
43.9
FMO Lag3
3.39
c
Gated w/in alive single cells
CD19
15.4
CD45.1
2.62
CD62L
CD44
0
62.1
0.5
37.4
Naive
Central Memory
Effector Memory
% w/in OTI T cells
**
d
# of ALL cells
***
e
% of NGFR+ ALL (w/in ALL)

Supplementary Figure 12. Adoptive transfer of OT-I T cells in OVA-ALL-injected CTRL, IFN and non-tumor bearing C57Bl/6 mice. a, Absolute numbers (mean ± SEM) of OVA-ALL in the PB of CTRL (n=7) and IFN (n=7) mice at the indicated time upon OT-I adoptive transfer. Each dot represents a mouse. **b**, Gating strategy and percentage of Lag3 positive OT-I T cells in BM and spleen of IFN (n= 7), CTRL (n=7) and non tumor-bearing (C57Bl/6 no tumor, n=3) mice, 3 days upon adoptive transfer of OT-I T cells. Each dot represents a mouse. Adoptively transferred OT-I T cells (CD45.2+) are identified by first excluding OVA-ALL cells by gating on CD19-CD8+ T cells. CD45.1 and CD45.2 markers are used to distinguish among radio-resistant recipient-derived CD8+ T cells (CD45.1+), donor-derived CD8+ T cells (CD45.1+CD45.2+) and adoptively transferred OT-I T cells (CD45.2+). In non tumor-bearing mice, adoptively transferred OT-I T cells are defined as CD8+ T cells and are distinguished from CD8+ recipient-derived T cells (CD45.1+) by gating on CD45.2+ CD8+ T cells. **c**, Gating strategy and percentage (mean ± SEM) of naïve (CD44-CD62L+), central memory (CD44+CD62L+) and effector memory (CD44+CD62L-) cells within OT-I T cells in the BM and spleen of mice from Fig. 7g, 3 days upon adoptive transfer. OT-I T cells are identified as described in (**b**, OVA-ALL are first excluded by gating on CD19-CD8+ T cells). **p<0.01, nonparametric combination test. **d**, **e**, Absolute numbers of ALL (**d**) (mean ± SEM) and NGFR expression on ALL (**e**) in BM and spleen from mice shown in Fig. 7g at 3 days upon adoptive transfer of OT-I T cells. Each dot represents a mouse. **p<0.01, ***p<0.001, Mann-Whitney (**d**).

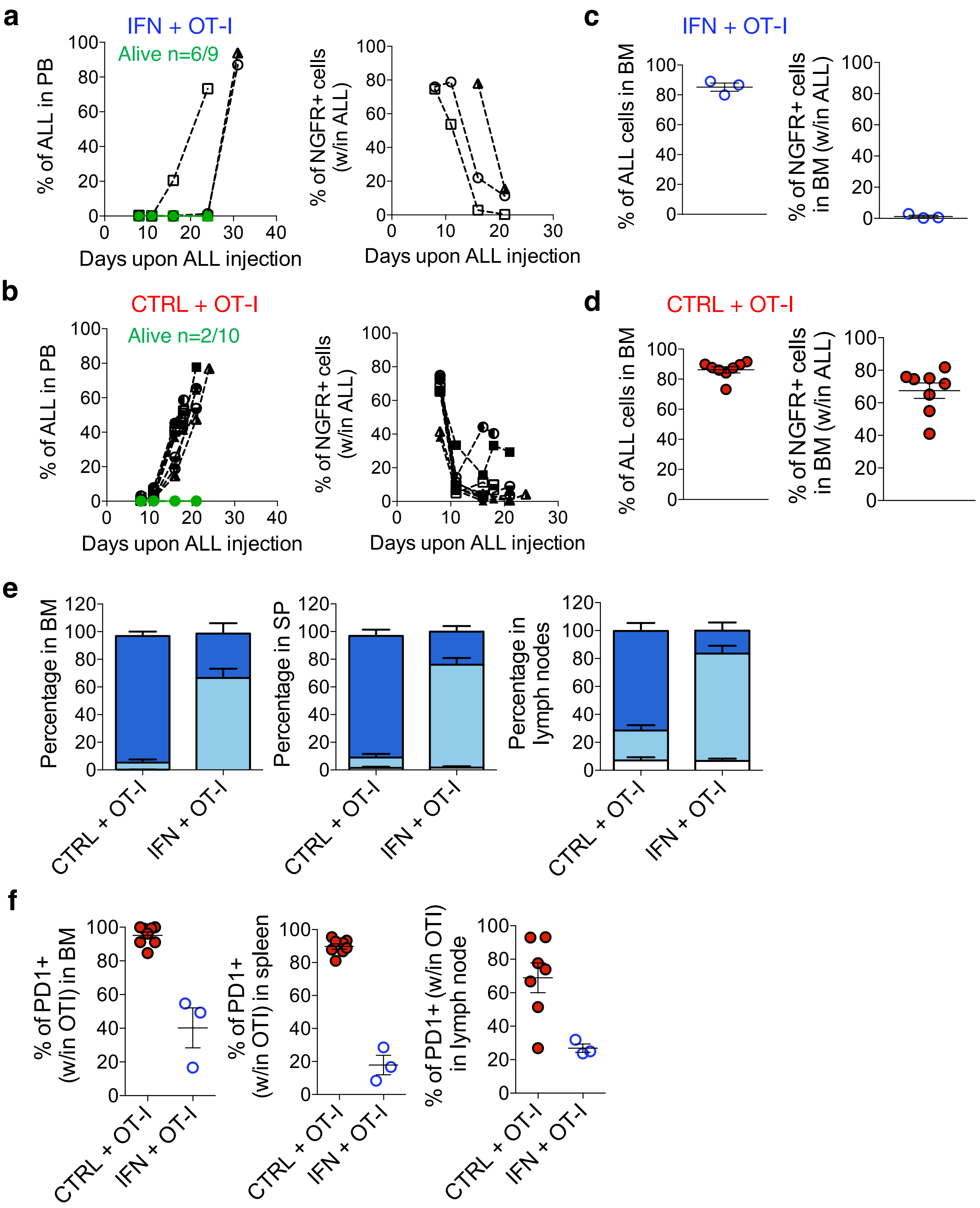
a
IFN + OT-I
Alive n=6/9
% of ALL in PB
Days upon ALL injection
% of NGFR+ cells (w/in ALL)
Days upon ALL injection
b
CTRL + OT-I
Alive n=2/10
% of ALL in PB
Days upon ALL injection
% of NGFR+ cells (w/in ALL)
Days upon ALL injection
c
IFN + OT-I
% of ALL cells in BM
% of NGFR+ cells in BM (w/in ALL)
d
CTRL + OT-I
% of ALL cells in BM
% of NGFR+ cells in BM (w/in ALL)
e
Percentage in BM
Percentage in SP
Percentage in lymph nodes
CTRL + OT-I
IFN + OT-I
f
% of PD1+ (w/in OTI) in BM
% of PD1+ (w/in OTI) in spleen
% of PD1+ (w/in OTI) in lymph node
CTRL + OT-I
IFN + OT-I

Supplementary Figure 13. Adoptively transferred OT-I T cells expand and contain OVA-ALL in IFN mice. **a**, **b**, **c**, **d**, Percentage (mean ± SEM) of ALL cells and expression of the NGFR marker on ALL cells in the PB (**a**, **b**) and BM (**c**, **d**) of IFN + OT-I (n=9, **a**, **c**) and CTRL + OT-I (n=10, **b**, **d**) mice. Long-term surviving mice are shown in green. Note that NGFR expression is not shown for surviving mice that eradicate leukemia. Each mouse is represented as single dot (**b**, **d**) or line (**a**, **b**). **e**, **f**, Percentage (mean ± SEM) of naive (CD62L+CD44-), central memory (CD62L+CD44+) and effector memory (CD62L-CD44+) OT-I T cells in the BM, spleen and lymph nodes of euthanized tumor-bearing mice from (**a**, **b**, **c**, **d**, CTRL + OT-I, n=8; IFN + OT-I, n=3). **f**, Percentage (mean ± SEM) of PD1 expression on OT-I T cells present in the BM, spleen and lymph nodes of mice shown in (**e**). Each dot represents a mouse.

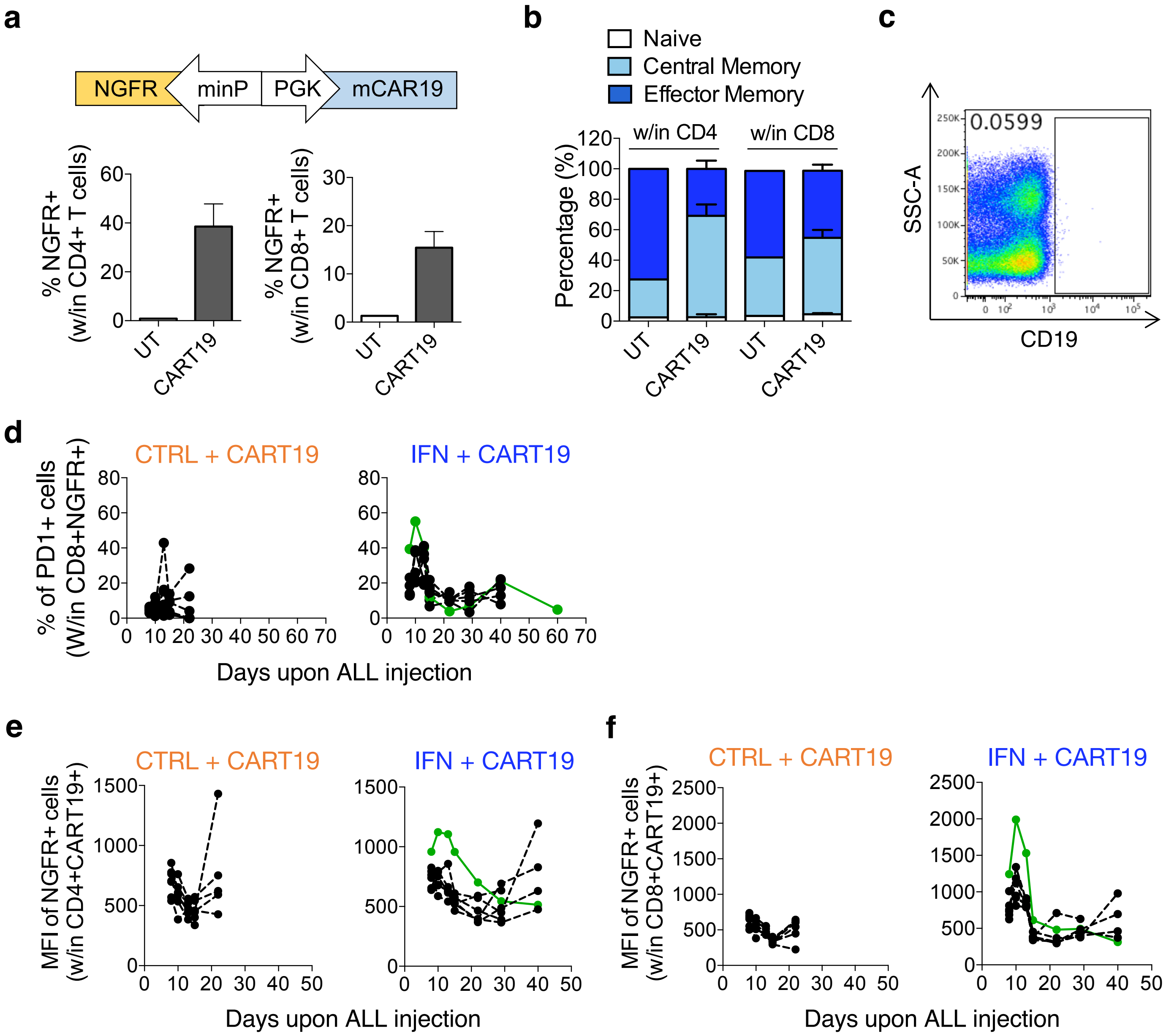

a
NGFR
minP
PGK
mCAR19
% NGFR+ (w/in CD4+ T cells)
% NGFR+ (w/in CD8+ T cells)
UT
CART19
b
Naive
Central Memory
Effector Memory
w/in CD4
w/in CD8
Percentage (%)
c
0.0599
SSC-A
CD19
d
CTRL + CART19
IFN + CART19
% of PD1+ cells (W/in CD8+NGFR+)
Days upon ALL injection
e
MFI of NGFR+ cells (w/in CD4+CART19+)
Days upon ALL injection
f
MFI of NGFR+ cells (w/in CD8+CART19+)
Days upon ALL injection

Supplementary Figure 14. IFN gene therapy boosts the activation of CART19 cells. a, Schematic representation of the bidirectional LV used to transduced mouse T cells and percentage of NGFR expression in un-transduced (UT) and transduced (CART19) CD4 and CD8 T cells prior to infusion in the mice from Fig. 8b. **b**, Percentage of naïve (CD62L+CD44+), central memory (CD62L+CD44+) and effector memory (CD62L-CD44+) UT or CART19 T cells prior to infusion in the mice from Fig. 8b. **c**, Representative flow cytofluorimetric plot of the IFN + CART19 surviving mouse from Fig. 8b and showing B cell aplasia in the PB. **d**, Percentage overtime (mean ± SEM) of PD1 expression on CD8+NGFR+ CART19 cells from CTRL + CART19 (n=7) and IFN + CART19 (n=7) mice. Long-term surviving mice are shown in green. Each line represents a mouse. **e**, **f**, Level (mean fluorescent intensity, MFI) of NGFR expression overtime on CD4+ (**e**) and CD8+ (**f**) CART19 cells of mice from (**d**). Long-term surviving mice are shown in green. Each line represents a mouse.

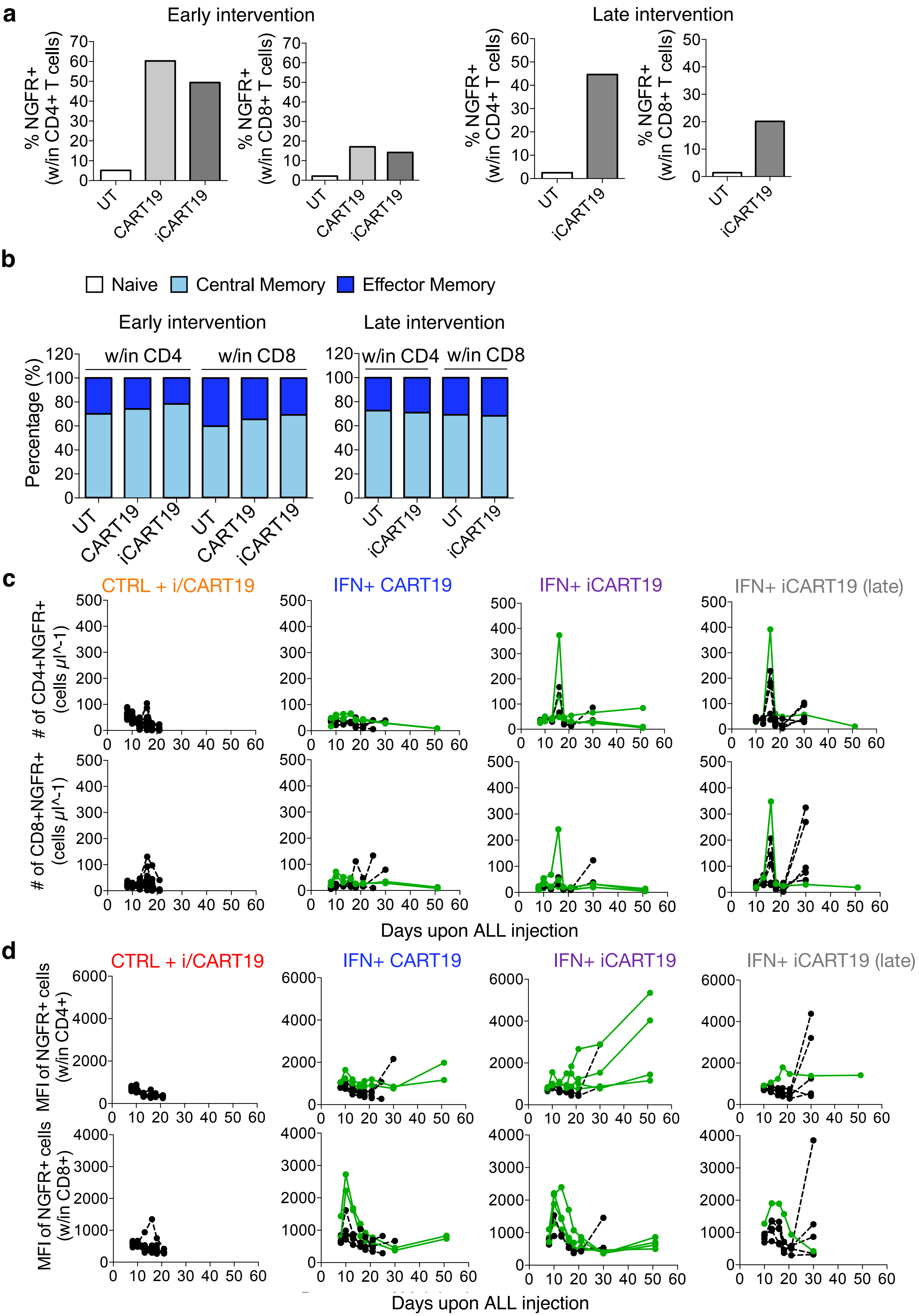
a
Early intervention
Late intervention
% NGFR+ (w/in CD4+ T cells)
% NGFR+ (w/in CD8+ T cells)
UT
CART19
iCART19
b
Naive
Central Memory
Effector Memory
Early intervention
Late intervention
w/in CD4
w/in CD8
Percentage (%)
c
CTRL + i/CART19
IFN+ CART19
IFN+ iCART19
IFN+ iCART19 (late)
# of CD4+NGFR+ (cells μl^-1)
# of CD8+NGFR+ (cells μl^-1)
Days upon ALL injection
d
CTRL + i/CART19
IFN+ CART19
IFN+ iCART19
IFN+ iCART19 (late)
MFI of NGFR+ cells (w/in CD4+)
MFI of NGFR+ cells (w/in CD8+)
Days upon ALL injection

Supplementary Figure 15. IFN gene therapy boosts the activation of iCART19 cells. a, Percentage of NGFR expression in un-transduced (UT) and transduced (CART19) CD4 and CD8 T cells prior to infusion in the mice from Fig. 8e. **b**, Percentage of naïve (CD62L+CD44+), central memory (CD62L+CD44+) and effector memory (CD62L-CD44+) UT or CART19 T cells prior to infusion in the mice from Fig. 8e. **c**, Absolute numbers (mean ± SEM) of CD4 (top) and CD8 (bottom) NGFR+ CART19 cells from CTRL + CART19 (n=7), IFN + CART19 (n=6), CTRL + iCART19 (n=7), IFN + iCART19 (n=6) and IFN + iCART19 late (n=6, late intervention trial) mice. Long-term surviving mice are shown in green. Each line represents a mouse. **d**, Level (mean fluorescent intensity, MFI) of NGFR expression overtime on CD4+ (top) and CD8+ (bottom) CART19 cells of mice from (**c**). Long-term surviving mice are shown in green. Each line represents a mouse.

		Antigen reactivity (tested in vitro by γ-IFN-ELISPOT)				
**Mouse**	**Group**	**OVA**	**tTA**	**OFP**	**NGFR**	**Re-challenge with OVA+/OVA- mixed ALL**
C4-58 (Exp. Fig 5a)	CTRL + IgG	✓	✓	✓		Alive
A0-58 (Exp. Fig 5a)	CTRL + CTLA4	✓	✓			Alive
B3-58 (Exp. Fig 5a)	CTRL + CTLA4	✓	✓			Alive
A2-62 (Exp. Fig 5a)	CTRL + CTLA4	✓	✓			Found dead
C1-73 (Exp. Fig 5d)	CTRL + CTLA4	✓	✓	✓		Not Done
E3-58 (Exp. Fig 5a)	IFN + IgG	✓			✓	Alive
F0-58 (Exp. Fig 5a)	IFN + IgG	*				Alive. * Note that circulating OVA-specific T cells were detected by cytofluorimetric analysis on blood samples collected overtime and stained with the OVA-specific pentamer reagent
F3-62 (Exp. Fig 5a)	IFN + IgG	✓		✓		Euthanized at 23 days upon re-challenge. The mouse shows the outgrowth of NGFR negative OFP negative ALL cells
D3-77 (Exp. Fig 4f)	IFN	✓	✓			Not Done
F4-77 (Exp. Fig 4f)	IFN	✓	✓			Not Done
E1-58 (Exp. Fig 5a)	IFN + CTLA4	✓	✓	✓	✓	Alive
G1-58 (Exp. Fig 5a)	IFN + CTLA4	✓	✓	✓		Alive
E4-62 (Exp. Fig 5a)	IFN + CTLA4	✓	✓			Alive
F0-62 (Exp. Fig 5a)	IFN + CTLA4	✓			✓	Euthanized at 23 days upon re-challenge. The mouse shows the outgrowth of NGFR negative ALL cells. Within splenic CD8+ T lymphocytes, 10% are OVA-specific T cells.
F1-62 (Exp. Fig 5a)	IFN + CTLA4	✓	✓	✓		Alive
E0-73 (Exp. Fig 5d)	IFN + CTLA4	✓	✓			Not Done
F2-73 (Exp. Fig 5d	IFN + CTLA4	✓	✓			Not Done
F3-73 (Exp. Fig 5d	IFN + CTLA4	✓	✓		✓	Not Done

Supplementary Figure 16. Long-term surviving mice shows immune reactivity toward multiple surrogate TSA. The table summarizes the reactivity of PBMC from surviving mice shown in Fig. 6f (IFN, n=2), Fig. 7a (CTRL, n=1; CTRL + aCTLA4, n=3; IFN, n=3; IFN + aCTLA4, n=5), Fig. 7d (CTRL + aCTLA4, n=1; IFN + aCTLA4, n=3) and tested by IFNγ-ELISPOT (13 days upon tumor injection in Fig 6f; 51 days upon OVA-ALL injection (first challenge) in Fig. 7a; 19 days upon tumor injection in Fig. 7d). Target EL4 cells transduced with the PGK-tTA or PGK-OFP LVs or with the NGFR-CD20 or NGFR-OVA BdLVs are used for ex-vivo PBMC re-stimulation. Surviving mice from Fig. 7a are then re-challenge with a mix of OVA-ALL and parental ALL cells (mixed at 1:1 ratio). Results of tumor re-challenge and of BM and spleen examinations on euthanized mice are reported.

Supplementary Table 1. Shown is the list of antibodies used.

Antibody	Fluorochrome	Dilution	Clone	Company	Code
Annexin V	PB	1:10		Biolegend	640918
CD11b	FITC	1:200	M1/70	BD	553310
CD11b	APC	1:100	M1/70	BD	553312
CD11b	PE	1:100	M1/70	BD	553311
CD11b	APC-Cy7	1:100	M1/70	Biolegend	101226
CD11b	PE	1:100	M1/70	BD	562317
CD11c	PeCy7	1:100	N418	eBioscience	25-0114-82
CD172a	APC	1:100	P84	eBioscience	17-1721-80
CD19	PE	1:200	1D3	BD	553786
CD19	PB	1:100	6D5	Biolegend	115526
CD19	PeCy7	1:100	6D5	Biolegend	115520
CD19	APC	1:200	1D3	BD	550992
CD19	FITC	1:400	1D3	BD	553785
CD25	APC	1:100	PC61	BD	557192
CD27	PeCy7	1:100	LG-7F9	eBioscience	25-0271-80
CD271	Alexa647	1:50	C40-1457	BD	560326
CD279	APC	1:100	J43	BD	562671
CD279	PB	1:100	29F.1A12	Biolegend	135202
CD223	PE	1:100	C9B7W	BD	552380
CD3	PB	1:100	17A2	Biolegend	100214
CD314	Biotin	1:100	A10	eBioscience	13-5872-82
CD335	PE	1:100	29A1.4	Biolegend	137604
CD3e	FITC	1:200	145-2C11	BD	553062
CD3e	PE	1:50	145-2C11	BD	553063
CD4	PeCy7	1:200	GK1.5	eBioscience	25-0041-82
CD4	PB	1:100	RM4-5	BD	558107
CD4	PE	1:200	RM4-5	BD	553049
CD44	APC	1:200	IM7	BD	559250
CD44	BV421	1:100	IM7	BD	563970
CD44	FITC	1:250	IM7	BD	553133
CD45.1	APC780	1:300	A20	eBioscience	47-0453-82
CD45.1	FITC	1:100	A20	BD	553775
CD45.1	PeCy7	1:100	A20	Biolegend	110730
CD45.2	FITC	1:100	104	BD	553772
CD45.2	PE	1:100	104	Biolegend	109807
CD45.2	PerCP-Cy5.5	1:100	104	BD	552950

CD45.2	PB	1:100	104	Biolegend	109820
B220	PE	1:100	RA3-6B2	BD	553090
CD49b	PerCPCy5.5	1:100	DX5	Biolegend	108916
CD62L	PE	1:100	MEL-14	BD	553151
CD8a	FITC	1:100	53-6.7	BD	553030
CD8a	PB	1:200	53-6.7	BD	558106
CD8a	PE	1:200	53-6.7	BD	533032
CD8a	PeCy7	1:200	53-6.7	BD	552877
CD8a	APC780	1:150	53-6.7	eBioscience	47-0081-82
CD20	APCH7		LT20	Miltenyi	130-096-640
F4/80	APC	1:100	A3-1	Biorad	MCA497CP CT
FOXP3	PE	1:100	FJK-16s	eBioscience	12-5773-82
I-A/I-E	APC-Cy7	1:100	M5/114.15 .2	Biolegend	107628
I-Ab	PerCP-Cy5.5	1:100	AF6.120.1	Biolegend	116416
IFN-g	APC	1:100	XMG1.2	Biolegend	505810
Ki67	Alexa647	1:10	B56	BD	558615
Ki67	PerCP-Cy5.5	1:10	B56	BD	561284
Ly-6C	APC-Cy7	1:100	HK1.4	Biolegend	128026
Ly-6G	PE	1:200	RB6-8C5	eBioscience	12-5931-83
NGFR	APC		ME20.4-1.H4	Miltenyi	130-091-884
NK.1.1	FITC	1:100	PK136	BD	553164
NK-1.1	PB	1:100	PK136	Biolegend	108722
Streptavidi n	Pacific orange	1:50		Invitrogen	532365
Ter119	PE	1:500	Ter-119	BD	553673
TIM3	APC	1:100	8B.2C12	eBioscience	17-5871-80

Supplementary Table 2. Results of statistical analysis performed on Fig. 1c Analysis performed using Nonparametric methods for Longitudinal Data in Factorial Experiments (see Methods section for details). We let Group 1 = CTRL, Group 2 = CTRL + CTLA4, Group 3 = IFN, Group 4 = IFN + CTLA4. The effects of group and time on growth, as well as their interaction, have been evaluated in a two-way nonparametric ANOVA-type framework. From the analysis, we can conclude that group and time effects are significant, while groups' dynamics are similar over time.

ANOVA-Type statistic			
	Statistic	df	p-value
Group	22.6818	2.4406	0.0000
Time	225.9886	1.7873	0.0000
Group:Time	1.5356	3.8203	0.1913

<u>Pairwise comparisons:</u>

Group 1 vs group 2: Pairwise comparison. Full factorial model has been estimated. After correction we observe only a significant effect of time. P-values adjusted by multiplicity are reported.

ANOVA-Type statistics					
			not corrected	6 comparisons	3 comparisons
	Statistic	df	p-value	p-value	p-value
Group	0.2096	1.0000	0.6471	1	1
Time	160.9945	1.9862	0.0000	0.0000	0.0000
Group:Time	0.2990	1.9862	0.7400	1	1

Group 1 vs group 3: Pairwise comparison. Full factorial model has been estimated. After correction we observe a significant group and time effect. P-values adjusted by multiplicity are reported. Groups' dynamics are similar over time

ANOVA-Type statistics					
			not corrected	6comparisons	3comparisons
	Statistic	df	p-value	p-value	p-value
Group	8.288586	1	0.0040	0.0239	0.0120
Time	121.2533	1.754652	0.0000	0.0000	0.0000
Group:Time	0.024141	1.754652	0.9647	1	1

Group 1 vs group 4. Pairwise comparison. Full factorial model has been estimated. After correction we observe a significant group and time effect. P-values adjusted by multiplicity are reported. Groups' dynamics are similar over time.

ANOVA-Type statistics				
			not corrected	6 comparisons
	Statistic	df	p-value	p-value
Group	84.65756	1	0.0000	0.0000
Time	64.444	1.724626	0.0000	0.0000
Group:Time	0.263118	1.724626	0.7355	1

Group 2 vs group 3: Pairwise comparison. Full factorial model has been estimated. After correction we observe a significant group and time effect. P-values adjusted by multiplicity are reported. Groups' dynamics are similar over time.

ANOVA-Type statistics					
			not corrected	6comparisons	3comparisons
	Statistic	df	p-value	p-value	p-value
Group	8.074028	1	0.0045	0.0269	0.0135
Time	177.8822	1.640893	0.0000	0.0000	0.0000
Group:Time	0.127261	1.640893	0.8404	1	1

Group 2 vs group 4: Pairwise comparison. Full factorial model has been estimated. After correction we observe a significant group and time effect. P-values adjusted by multiplicity are reported. Groups' dynamics are similar over time

ANOVA-Type statistics				
			not corrected	6 comparisons
	Statistic	df	p-value	p-value
Group	72.43085	1	0.0000	0.0000
Time	78.76083	1.515773	0.0000	0.0000
Group:Time	0.978513	1.515773	0.3559	1

Group 3 vs group 4: Pairwise comparison. Full factorial model has been estimated. After correction we observe a significant group and time effect. P-values adjusted by multiplicity are reported. Groups' dynamics are similar over time.

ANOVA-Type statistics					
			not corrected	6 comparisons	3 comparisons
	Statistic	df	p-value	p-value	p-value
Group	12.20105	1	0.0005	0.0029	0.0014
Time	80.77027	1.497091	0.0000	0.0000	0.0000
Group:Time	0.633509	1.497091	0.4867	1	1

Supplementary Table 3. Results of statistical analysis performed on Fig. 1d. Analysis performed using Nonparametric methods for Longitudinal Data in Factorial Experiments, by employing the package nparLD (see Methods section for details) referring to figure 1d. We let Group 1 = CTRL, Group 2 = IFN. We consider statistical significance at level alpha = 0.05. Due to the aforementioned requirement for each mouse to be observed at all considered times, we omit mouse 2 (IFN group) from the analysis. An alternative approach which imputed the missing value with the mean of the previous and subsequent values for that mouse yielded similar results for the analysis. Hence, we decide to exclude the mouse altogether from the analysis. Nonparametric method for Longitudinal Data in Factorial Experiments has been applied. The effects of group and time on growth, as well as their interaction, have been evaluated in a two-way nonparametric ANOVA-type framework. We observe a significant effect of group and time but the interaction effect was not significant, meaning that the dynamics of the two groups are similar through time.

ANOVA-Type statistic			
	Statistic	df	p-value
Group	14.8094	1.0000	0.0001
Time	81.0775	1.1720	0.0000
Group:Time	2.2470	1.1720	0.1295

Supplementary Table 4. Results of statistical analysis performed on Fig. 2d. Analysis performed using Nonparametric methods for Longitudinal Data in Factorial Experiments, by employing the package nparLD (see Methods section for details). We let Group 1 = CTRL, Group 2 = IFN Depleted, Group 3 = IFN. We consider statistical significance at level alpha = 0.05. Due to the aforementioned requirement for each mouse to be observed at all considered times, we omit mouse 2 and mouse 5 (IFN Depleted group) from the analysis, as they their values are not observed at the last time point. Nonparametric methods for Longitudinal Data in Factorial Experiments. The effects of group and time on growth, as well as their interaction, have been evaluated in a two-way nonparametric ANOVA-type framework. From the analysis, we can conclude that there are significant group and time effects, as well as a significant interaction effect between time and each group, meaning that the groups' dynamics are not all equal.

ANOVA-Type Statistic		
	Statistic	p-value
Group	12.608	0.000
Time	213.942	0.000
Group by Time	6.602	0.002

Pairwise comparisons:

Group 1 vs group 2: Pairwise comparison. Full factorial model has been estimated. We observe only a significant effect for difference over time. The two groups increase over time but there is no evidence for a significant difference between them. P-values adjusted by multiplicity are reported.

ANOVA-Type Statistic		
	Statistic	p-value
Group	0.754	1.000
Time	671.710	0.000
Group by Time	1.374	0.740

Group 1 vs. Group 3: Pairwise comparison. Full factorial model has been estimated. We observe a significant group and time effect. This means that the two groups differ in level but they share the same dynamics over time. P-values adjusted by multiplicity are reported.

ANOVA-Type Statistic		
	Statistic	p-value
Group	21.414	0.000
Time	82.097	0.000
Group by Time	3.936	0.109

Group 2 vs Group 3: Pairwise comparison. Full factorial model has been estimated. We observe a significant group and time effect. This means that the two groups differ in level but they share the same dynamics over time. P-values adjusted by multiplicity are reported.

ANOVA-Type Statistic		
	Statistic	p-value
Group	11.018	0.003
Time	71.081	0.000
Group by Time	4.675	0.063

Supplementary Table 5. Results of statistical analysis performed on Fig. 7a. We performed a k-sample Log-rank test for the differences between the estimated survival curves (by Kaplan-Meier estimation method for the survival curves) for the four Groups CTRL, CTRL + CTLA4, IFN, IFN + CTLA4. All the subsequent p-values for the pair-wise comparisons are adjusted for multiplicity. We performed three planned comparisons between the control group and all other groups.

Pairwise comparisons:

CTRL vs. CTRL + CTLA4: The hypothesis of equality between the two survival curves can be rejected (adjusted p-value =0.04867367)

CTRL vs. IFN: The hypothesis of equality between the two survival curves can be rejected (adjusted p-value = 0.04557663)

CTRL vs. IFN + CTLA4: The hypothesis of equality between the two survival curves can be rejected here (adjusted p-value = 0.0006989215)

Supplementary Table 6. Results of statistical analysis performed on Fig. 7b. Group 1 = CTRL, Group 2 = IFN, Group 3 = CTRL +CTLA4, Group 4 = IFN+CTLA4. Nonparametric methods for Longitudinal Data in Factorial Experiments. The effects of group and time on growth, as well as their interaction, have been evaluated in a two-way nonparametric ANOVA-type framework. From the analysis, we can conclude that all the considered effects are significant, meaning that the groups' dynamics are not all equal over time.

ANOVA-Type Statistic		
	Statistic	p-value
Group	7.566	0.000
Time	61.645	0.000
Group by Time	2.556	0.024

Pairwise comparisons:

Group 1 vs Group 2: Pairwise comparison. Full factorial model has been estimated. We observe a significant effect of group and time effects. The growth rate is similar between the two groups since the interaction term is not significant. P-values adjusted by multiplicity are reported.

ANOVA-Type Statistic		
	Statistic	p-value
Group	7.648	0.034
Time	28.132	0.000
Group by Time	4.768	0.068

Group 1 vs Group 3: Pairwise comparison. Full factorial model has been estimated. We observe significant group and time effects. The interaction effect is not significant and thus we can conclude that both groups exhibit similar dynamics through time. P-values adjusted by multiplicity are reported.

ANOVA-Type Statistic		
	Statistic	p-value
Group	12.371	0.003
Time	14.345	0.000
Group by Time	0.913	1.000

Group 1 vs Group 4: Pairwise comparison. Full factorial model has been estimated. We observe significant difference between the two groups and throughout time. However, the interaction effect is not significant and thus we can conclude that both groups exhibit similar dynamics through time. P-values adjusted by multiplicity are reported.

ANOVA-Type Statistic		
	Statistic	p-value
Group	20.339	0.000
Time	26.825	0.000
Group by Time	3.700	0.158

Group 2 vs Group 3: Pairwise comparison. We find a significant effect of time, but group variable as well as the interaction group by time did not play a significant role in modulating the outcome variable. Thus, we can conclude that the two groups develop in the same way and do not exhibit significant differences at any stage. P-values adjusted by multiplicity are reported.

ANOVA-Type Statistic		
	Statistic	p-value
Group	0.235	1.000
Time	33.481	0.000
Group by Time	2.403	0.556

Group 2 vs Group 4: Pairwise comparison. We observe only a significant difference between the times. Thus, we can conclude that the two groups develop the same way

and do not exhibit significant differences at any stage. P-values adjusted by multiplicity are reported.

ANOVA-Type Statistic		
	Statistic	p-value
Group	2.042	0.918
Time	50.609	0.000
Group by Time	0.203	1.000

Group 3 vs Group 4: Pairwise comparison. Full factorial model has been estimated. We observe only a significant effect of time factor. Hence, the two groups develop the same way and do not exhibit significant differences at any stage. P-values adjusted by multiplicity are reported.

ANOVA-Type Statistic		
	Statistic	p-value
Group	1.135	1.000
Time	31.976	0.000
Group by Time	3.147	0.281

Supplementary Table 7. Results of statistical analysis performed on Fig. 7i.

We performed a k-sample Log-rank test for the differences between the estimated survival curves (by Kaplan-Meier estimation method for the survival curves) for the three Groups CTRL, CTRL + OTI, IFN + OT-I. All the subsequent p-values for the pair-wise comparisons are adjusted for multiplicity.

<u>Pairwise comparisons:</u>

CTRL vs. CTRL + OTI: The hypothesis of equality between the two survival curves cannot be rejected (adjusted p-value = 0.1513032)

CTRL vs. IFN + OTI: The hypothesis of equality between the two survival curves can be rejected (adjusted p-value = 9.181594e-05)

CTRL + OTI vs. IFN + OTI: The hypothesis of equality between the two survival curves can be rejected (adjusted p-value = 0.03864594)

Supplementary Table 8. Results of statistical analysis performed on Fig. 8b.

Group 1 = CTRL + CTRLT, Group 2 = CTRL + CART19, Group 3 = IFN + CTRLT, Group 4 = IFN + CART19. Nonparametric methods for Longitudinal Data in Factorial Experiments. The effects of group and time on growth, as well as their interaction, have been evaluated in a two-way nonparametric ANOVA-type framework. We conclude that all the considered effects (group, time and group by time interaction) are significant.

ANOVA-Type Statistic		
	Statistic	p-value
Group	68.508	0.000
Time	112.472	0.000
Group by Time	9.812	0.000

<u>Pairwise comparisons:</u>

Pairwise comparison (Group 1 vs group 2). Full factorial model has been estimated. We observe only a significant effect of time. Group effect as well as the interaction term are not significant. Thus, we can conclude that the two groups develop in the same way and do not exhibit significant differences at any stage. P-values adjusted by multiplicity are reported.

ANOVA-Type Statistic (ATS)		
	Statistic	p-value
Group	0.919	1.000
Time	184.497	0.000
Group by Time	0.858	1.000

Pairwise comparison (Group 1 vs group 3). Full factorial model has been estimated. We observe a significant difference between the groups and throughout time (significant group and time effects). We conclude that the growth rate is similar between the two groups. P-values adjusted by multiplicity are reported.

ANOVA-Type Statistic (ATS)		
	Statistic	p-value
Group	43.904	0.000
Time	228.729	0.000
Group by Time	1.254	1.000

Pairwise comparison (Group 1 vs group 4). Full factorial model has been estimated. We observe a significant difference between the groups and throughout time (significant group and time effects). After adjustment for multiplicity, the interaction effect is not significant. P-values adjusted by multiplicity are reported

ANOVA-Type Statistic (ATS)		
	Statistic	p-value
Group	100.269	0.000
Time	7.297	0.001
Group by Time	2.970	0.226

Pairwise comparison (Group 2 vs group 3). Full factorial model has been estimated. We observe a significant difference between the groups and throughout time (significant group and time effects). Since the interaction effect is not significant, we conclude that the growth rate is similar between the two groups. P-values adjusted by multiplicity are reported.

ANOVA-Type Statistic (ATS)		
	Statistic	p-value
Group	25.104	0.000
Time	268.864	0.000
Group by Time	1.165	1.000

Pairwise comparison (Group 2 vs group 4). Full factorial model has been estimated. All tested effects are significant (group, time and group by time interaction). P-values

adjusted by multiplicity are reported.

ANOVA-Type Statistic (ATS)		
	Statistic	p-value
Group	70.802	0.000
Time	10.912	0.000
Group by Time	5.554	0.010

Pairwise comparison (Group 3 vs group 4). Full factorial model has been estimated. We observe a significant effect of time and group. After adjustment for multiplicity, the interaction effect is not significant. P-values adjusted by multiplicity are reported.

ANOVA-Type Statistic (ATS)		
	Statistic	p-value
Group	20.669	0.000
Time	7.684	0.002
Group by Time	3.748	0.124

Supplementary Table 9. Results of statistical analysis performed on Fig. 8e. Group 1 = CTRL + CTRLT, Group 2 = CTRL + CART19, Group 3 = CTRL + iCART19, Group 4 = IFN + CTRLT, Group 5 = IFN + CART19, Group 6 = IFN + iCART19. Nonparametric methods for Longitudinal Data in Factorial Experiments. The effects of group and time on growth, as well as their interaction, have been evaluated in a two-way nonparametric ANOVA-type framework. All the considered effects (group, time and time by group interaction) are significant.

ANOVA-Type Statistic (ATS):			
	Statistic	df	p-value
Group	25.569	2.625	0.000
Time	189.852	2.553	0.000
Group by Time	8.256	5.957	0.000

Pairwise comparisons:

Pairwise comparison (Group 1 vs group 2). Full factorial model has been estimated. We find only a significant effect of time. Group effect as well as the interaction term (after adjustment for multiplicity) are not significant. Thus, we can conclude that the two groups develop in the same way. P-values adjusted by multiplicity are reported.

ANOVA-Type Statistic (ATS)		
	Statistic	p-value
Group	2.418	0.720
Time	268.964	0.000
Group by Time	4.214	0.069

Pairwise comparison (Group 1 vs group 3). We find a significant effect of time and of the interaction term, indicating that the shape of the growth curves differed across groups (hence groups develop in a different way at some time points). P-values adjusted by multiplicity are reported.

ANOVA-Type Statistic (ATS)		
	Statistic	p-value
Group	1.490	1.000
Time	321.616	0.000
Group by Time	7.258	0.002

Pairwise comparison (Group 2 vs group 3). Full factorial model has been estimated. We find only a significant effect of time. Group and interaction terms are not significant. Thus, we can conclude that the two groups develop in the same way and do not exhibit significant differences at any stage. P-values adjusted by multiplicity are reported.

ANOVA-Type Statistic (ATS)		
	Statistic	p-value
Group	0.002	1.000
Time	207.671	0.000
Group by Time	0.804	1.000

Pairwise comparison (Group 5 vs group 6). Full factorial model has been estimated. We find only a significant effect of time. P-values adjusted by multiplicity are reported.

ANOVA-Type Statistic (ATS)		
	Statistic	p-value
Group	0.072	1.000
Time	8.247	0.000
Group by Time	0.942	1.000

Pairwise comparison (Group 2 vs group 5). Full factorial model has been estimated. From the analysis, we can conclude that all the considered effects (group, time and group by time interaction) are significant. P-values adjusted by multiplicity are reported.

ANOVA-Type Statistic (ATS)		
	Statistic	p-value
Group	23.722	0.000
Time	37.786	0.000
Group by Time	5.283	0.030

Pairwise comparison (Group 3 vs group 6). Full factorial model has been estimated. We conclude that all the considered effects (group, time and group by time interaction) are significant. P-values adjusted by multiplicity are reported.

ANOVA-Type Statistic (ATS)		
	Statistic	p-value
Group	67.813	0.000
Time	24.792	0.000
Group by Time	8.405	0.000

Supplementary Table 10. Results of statistical analysis performed on Fig. 8e.
Group 3 = CTRL + iCART19, Group 6 = IFN + iCART19, Group 8 = IFN + iCART19 (late). Nonparametric methods for Longitudinal Data in Factorial Experiments. The effects of group and time on growth, as well as their interaction, have been evaluated in a two-way nonparametric ANOVA-type framework. Only time points, common to all groups, have been considered. From the analysis, we can conclude that all the considered effects (group, time and group by time interaction) are significant.

ANOVA-Type Statistic (ATS):			
	Statistic	df	p-value
Group	35.401	1.825	0.000
Time	17.180	2.485	0.000
Group by Time	3.426	3.988	0.008

<u>Pairwise comparisons:</u>
Pairwise comparison (Group 3 and Group 8). From the analysis, we can conclude that all the considered effects (group, time and time by group interaction) are significant. P-values adjusted by multiplicity are reported.

ANOVA-Type Statistic (ATS)		
	Statistic	p-value
Group	62.752	0.000
Time	48.898	0.000
Group by Time	8.229	0.000

Pairwise comparison (Group 6 and Group 8). Full factorial model has been estimated. We find only a significant effect of time. Group and interaction effects are not significant. Thus, we conclude that the two groups develop in the same way and do not exhibit significant differences at any stage. P-values adjusted by multiplicity are reported.

ANOVA-Type Statistic (ATS)		
	Statistic	p-value
Group	2.695	0.201
Time	7.456	0.000
Group by Time	1.892	0.276

Supplementary Table 11. Results of statistical analysis performed on Fig. 8g.

We performed a k-sample Log-rank test for evaluating the differences among the estimated survival curves (by Kaplan-Meier estimation method for the survival curves) for the 4 groups CTRL + i/CART19, IFN + CART19, IFN + iCART19, IFN + iCART19 (late). All the subsequent p-values for the pair-wise comparisons are adjusted for multiplicity. Five comparisons in total have been considered.

Comparison	Adjusted *p*-value
CTRL + i/CART19 vs IFN + CART19	0.0000
CTRL + i/CART19 vs IFN + iCART19	0.0001
CTRL + i/CART19 vs IFN + iCART19 (late)	0.0001
IFN + CART19 vs IFN + iCART19	0.6326
IFN + CART19 vs IFN + iCART19 late	1.0000
